# Evaluating cardiac noise correction approaches for non-invasive electrophysiology of the human spinal cord

**DOI:** 10.1162/IMAG.a.938

**Published:** 2025-10-22

**Authors:** Emma Bailey, Birgit Nierula, Tilman Stephani, Burkhard Maess, Vadim V. Nikulin, Falk Eippert

**Affiliations:** Max Planck Research Group Pain Perception, Max Planck Institute for Human Cognitive and Brain Sciences, Leipzig, Germany; Research Group Neural Interactions and Dynamics, Department of Neurology, Max Planck Institute for Human Cognitive and Brain Sciences, Leipzig, Germany; Methods and Development Group Brain Networks, Max Planck Institute for Human Cognitive and Brain Sciences, Leipzig, Germany

**Keywords:** electrospinography, cardiac artefact, noise correction, spinal cord, human

## Abstract

The spinal cord is an important component of the central nervous system for the processing of sensorimotor information transmitted between the body and the brain. Electrospinography (ESG) is the most accessible non-invasive technique for recording spinal signals in humans, but the detrimental impact of physiological noise (mostly of cardiac nature) has prevented widespread adoption. Here, we aim to address this issue by examining various denoising algorithms for cardiac artefact reduction—including approaches based on principal component analysis (PCA), independent component analysis (ICA), signal space projection (SSP), canonical correlation analysis (CCA), and denoising separation of sources (DSS). We observed that in situations where large number of spinal electrodes are used, ICA and SSP offer the best results in terms of balancing the removal of noise and preserving neural information of interest. In cases where only a small number of electrodes are available, an approach based on PCA is deemed helpful. Finally, we also approached this issue from a signal-enhancement perspective by applying CCA and DSS directly to signals of interest, namely spinal somatosensory evoked potentials (SEPs, especially the N13 and N22 components in the cervical and lumbar spinal cord, respectively). We observed that in cases where extensive electrode arrays are used in the context of task-based designs, CCA reveals clear evoked spinal potentials even with single-trial resolution. Taken together, there are several appropriate algorithms for physiological noise removal and/or signal enhancement in ESG, rendering this an accessible and easy-to-use technique for non-invasive assessments of human spinal cord function.

## 1 Introduction

The spinal cord is an essential communication pathway between the brain and the peripheral nervous system and is of relevance for sensory, motor, and autonomic function (Hochman, 2007). Recent research in animal models has demonstrated that extensive somatosensory processing occurs in the dorsal horn of the spinal cord (Abraira et al., 2017; Paixão et al., 2019), yet direct investigations in humans remain rather scarce due to difficulties in non-invasively investigating this structure. There is, however, a pressing need for such recordings, not only to investigate human spinal cord function in health, but also to understand pathological changes that underlie conditions such as chronic pain, multiple sclerosis, and spinal cord injury (Ahuja et al., 2017; Ciccarelli et al., 2019; Kuner & Flor, 2017).

Of the currently available approaches for non-invasive recordings, functional magnetic resonance imaging of the human spinal cord (Kinany et al., 2023; Landelle et al., 2021) offers superior spatial resolution, but is fundamentally limited by its indirect nature (due to neurovascular coupling) and its coarse temporal resolution. These aspects have been addressed using magnetospinography (MSG) based on superconducting quantum interference devices (Adachi et al., 2021; Sumiya et al., 2017). Though successful, these systems are employed in less than a handful of laboratories worldwide, are costly to implement, and are not yet commercially available. Alternative MSG recordings based on optically pumped magnetometers (OPMs) have recently been proposed (Mardell et al., 2024; O’Neill et al., 2025; Spedden et al., 2024a, 2024b), yet this technology is currently only in a nascent stage. In contrast to these more recent approaches, there is substantial literature spanning several decades on the non-invasive recording of spinal cord somatosensory evoked potentials (SEPs) using readily available electrospinography technology (for reviews, see Cruccu et al., 2008; Mauguière, 2000; Yamada et al., 1980). Such a setup typically involves transcutaneous electrical stimulation of peripheral nerves and spinal recordings via surface electrodes placed on the neck or the lower back, depending on the responses of interest.

**Fig. 1.**
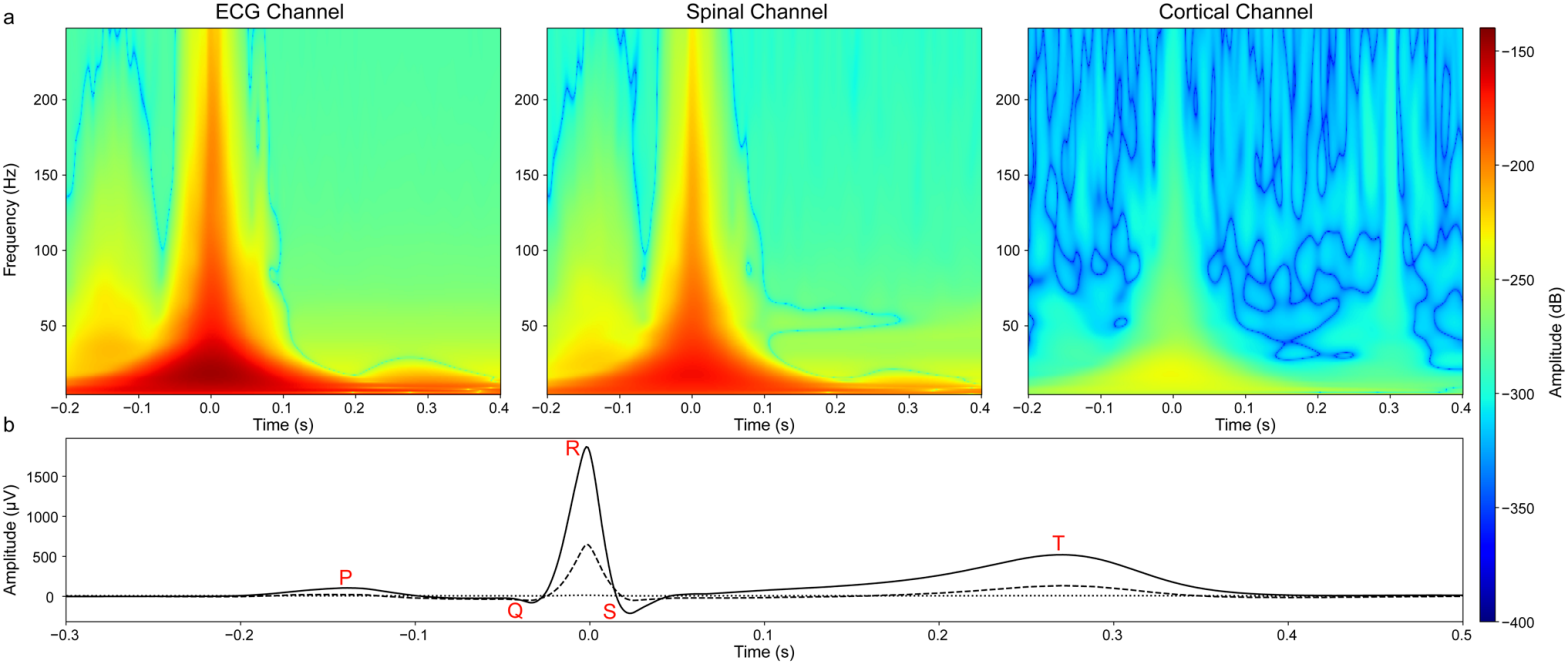
(a) Grand average (N = 36) time–frequency plots demonstrating the manifestation of the heartbeat in the ECG recording channel, with the corresponding effect of the heartbeat in a spinal (L1) and cortical (CPz) channel of interest. The amplitude difference between the spinal and cortical channels shows how detrimental the cardiac artefact is in spinal, as compared with cortical channels. (b) A corresponding time–domain depiction of the cardiac artefact in the ECG channel (solid), the spinal channel of interest (dashed), and the cortical channel of interest (dotted), with key components of the heartbeat shown in red lettering. In all plots, the R-peak occurs at 0 seconds.

However, in such electrospinographic (ESG) recordings, physiological noise of myogenic and especially cardiac origin is highly detrimental due to the proximity of the recording electrodes to the source of physiological noise, that is, the heart in the latter case (Cracco, 1973; Cracco et al., 1979; Jones & Small, 1978). In the cervical spinal cord, SEPs generally have an amplitude of approximately 1 to 2 microvolts (Cruccu et al., 2008), while the cardiac artefact reaches up to several hundred microvolts and has overlapping frequency content with SEPs. This detrimental influence is much reduced when standard electroencephalographic (EEG) recordings are performed, thus presenting a challenge unique to ESG recordings (Fig. 1).

The confounding influence of the cardiac artefact is traditionally mitigated in ESG studies via (i) employing cardiac-gated stimulation, (ii) averaging across large number of trials, or (iii) extensive high-pass filtering (e.g., at ~30 Hz). While effective, each of these approaches has drawbacks: (i) cardiac gating does not allow for studies where cardiac–somatosensory interactions are of interest (Al et al., 2020), (ii) the necessary number of trials to perform large-scale averaging is unsuitable in modern cognitive neuroscience paradigms (Gijsen et al., 2021), and (iii) high-pass filtering is undesirable in the case of resting-state recordings (Mantini et al., 2007; Wang et al., 2021). Evidently, there is need for an approach that reduces the detrimental impact of the cardiac artefact without imposing such limitations.

There are several approaches known to remove cardiac artefacts in other types of neurophysiological recordings, but to our knowledge, there has been no systematic investigation into the suitability of these methods for non-invasive ESG data. Here, we thus explored the ability of five different algorithms to reduce the impact of the cardiac artefact on ESG data. First, we adapted the principal component analysis optimal basis sets (PCA-OBS) approach pioneered by Niazy et al. (2005), which has previously been shown to work well for ESG data (Nierula et al., 2024), and can be applied even when data are recorded using limited number of, or even single, electrodes. Second, we employed independent component analysis (ICA), which is one of the most popular methods for removing artefacts and has been employed for cardiac artefact removal in both EEG data (Al et al., 2020) and invasively recorded spinal data (Wang et al., 2021). Third, we investigated the potential of signal space projection (SSP: Uusitalo & Ilmoniemi, 1997), a technique that has previously been implemented to remove cardiac artefacts from EEG and ESG data (Haumann et al., 2016; Nierula et al., 2024). Fourth, we tested the ability of canonical correlation analysis (CCA) to remove the cardiac artefact—an approach employed in both simultaneous EEG-fMRI and ESG studies in the past (Assecondi et al., 2009; Mehra et al., 2025). Finally, we examined the performance of denoising separation of sources (DSS) to remove the cardiac artefact, which has been shown to be effective for MEG data (De Cheveigné & Parra, 2014). Taken together, while all algorithms have been successfully applied in cortical recordings, their comparative efficacy for cardiac artefact removal in non-invasive surface recordings from the spinal cord remains unknown and is the focus of this investigation.

We also used both CCA and DSS to enhance task-evoked potentials in ESG data—an approach that has been used in previous ESG literature, as well as cortical EEG and MEG studies (Chander et al., 2022; De Cheveigné & Parra, 2014; Nierula et al., 2024; Stenner et al., 2025; Stephani et al., 2020; Waterstraat et al., 2015). This alternative analysis was performed in order to determine whether it might be possible to obtain reliable SEPs even in the absence of dedicated cardiac artefact correction, and whether additional benefits can be achieved through the combination of signal enhancement with dedicated cardiac artefact removal methods.

## 2 Methods

### 2.1 Data acquisition

The data used here come from recordings in 36 healthy right-handed volunteers (18 female), who received electrical mixed nerve stimulation of the median nerve at the left wrist and of the tibial nerve at the left ankle (Nierula et al., 2024). All participants provided written informed consent prior to participation and the study was approved by the ethics committee of the medical faculty at the University of Leipzig. Electrical stimulation consisted of a 0.2 ms square-wave pulse delivered by a constant-current stimulator (DS7A, Digitimer Ltd, Hertfordshire, UK) connected to a bipolar stimulation electrode (with a 25 mm electrode distance); one such setup was used for median nerve stimulation at the wrist and an identical setup was used for tibial nerve stimulation at the ankle. Stimulus intensities were adjusted on an individual level to be just above the motor threshold, but not deemed to be painful. Stimulation was provided with an inter-stimulus interval of 763 ms and a jitter of at most +/-50 ms. A total of 2000 stimuli were delivered in alternating blocks of 500 stimuli to either the median or tibial nerve. The presentation of stimuli (including breaks between blocks) took approximately 90 minutes.

**Fig. 2.**
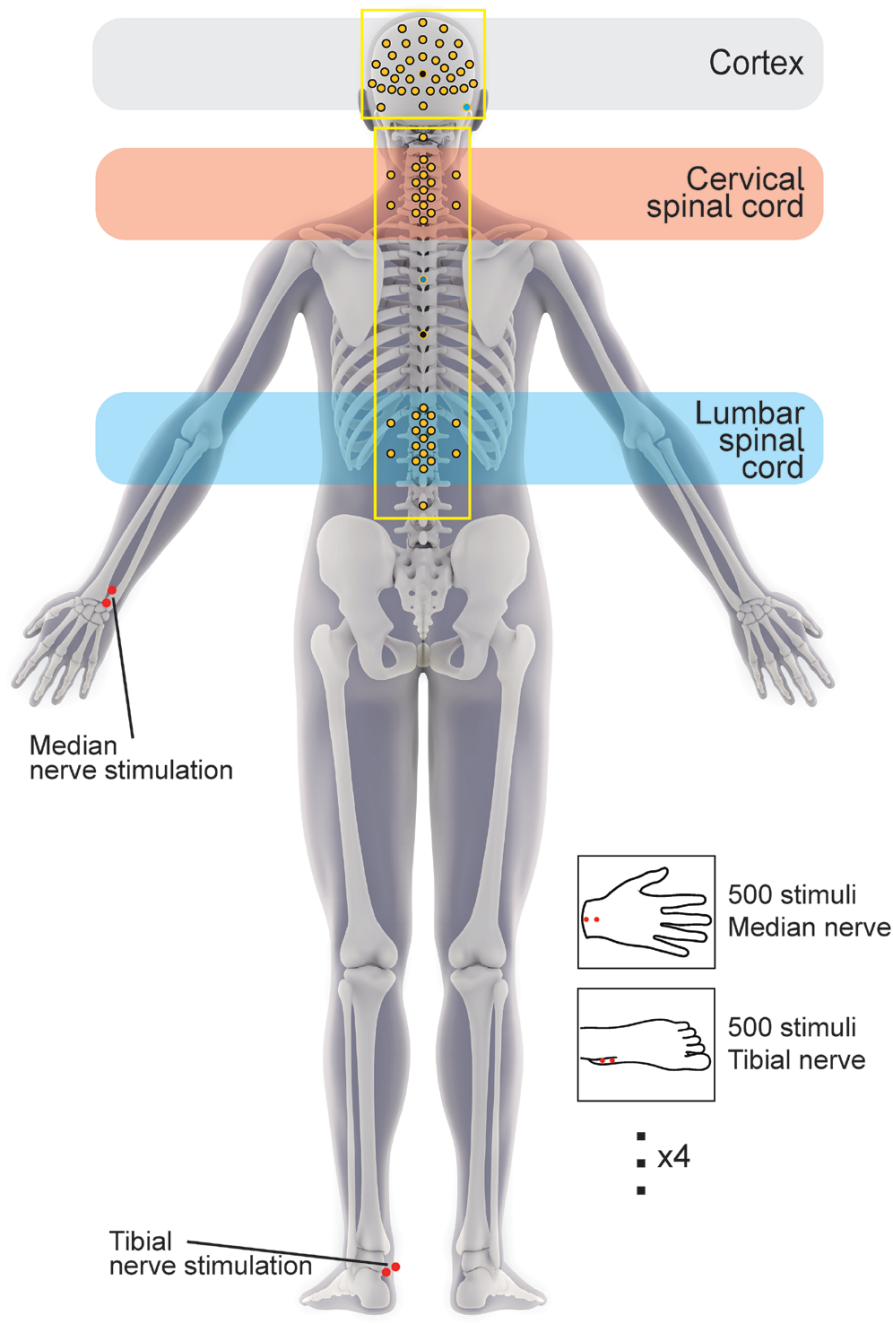
Recording electrode arrangement at the level of the cortex, cervical spinal cord, and lumbar spinal cord, alongside stimulating electrodes at the left wrist and left ankle, overlaid on a body image (Nerthuz, 2017). Inset is the block structure—for each participant, the starting condition (median or tibial nerve stimulation) was pseudo-randomised, and stimulation was thereafter applied in alternating blocks of 500 trials until 2000 trials per condition were achieved.

The publicly available dataset (https://openneuro.org/datasets/ds004388) includes simultaneously recorded electroencephalography (EEG), electrospinography (ESG), electroneurography (ENG), electromyography (EMG), electrocardiography (ECG), and respiratory information. For the purpose of this study, we focus on the ESG data with ECG data being used for assessing cardiac activity. For the ESG recordings, 2 separate patches of 17 electrodes each were placed on a participant’s neck and back: 1 patch was centred on the cervical spinal cord around an electrode placed over the spinous process of the 6th cervical vertebra and another patch was centred on the lumbar spinal cord around an electrode placed over the spinous process of the 1st lumbar vertebra (see Fig. 2). The electrodes were arranged along the midline of the vertebral column and extended laterally from there, with the ESG reference electrode being located between the cervical and lumbar spinal electrodes over the spinal process of the 6th thoracic vertebra; two ventral electrodes were also placed to enable anterior re-referencing (more details on patch construction are available in the original publication: Nierula et al., 2024). For median nerve stimulation, channels of interest were determined to be SC6 (the central cervical electrode located over the spinous process of the 6th cervical vertebra) as well as S6 and S14 (the immediately neighbouring electrodes directly above and below SC6 along the vertebral column). For tibial nerve stimulation, channels of interest were determined to be L1 (the central lumbar electrode located over the spinous process of the 1st lumbar vertebra) as well as S23 and S31 (the immediately neighbouring electrodes directly above and below L1 along the vertebral column). These channels represent midline channels in the array, and generally have the strongest somatosensory evoked potentials due to their positioning.

### 2.2 ESG preprocessing

**Fig. 3.**
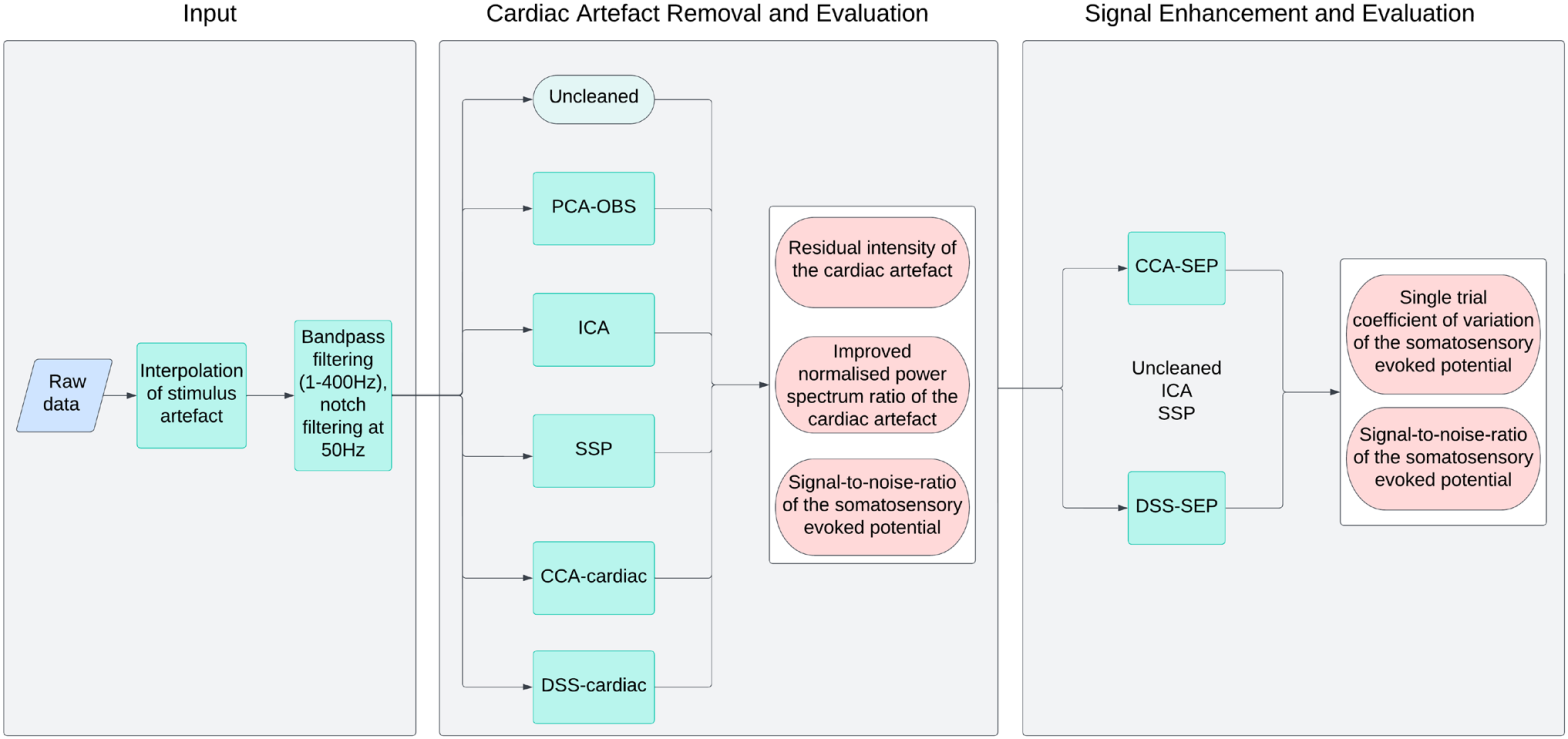
Overview of methods implementation. First (left panel), the input data are pre-processed to remove the stimulation artefact, bandpass filtered from 1 to 400 Hz, and notch filtered at 50 Hz to remove powerline noise. Data after this stage are referred to as Uncleaned. These Uncleaned data (middle panel) then have the cardiac artefact removed by either principal component analysis optimal basis sets (PCA-OBS), independent component analysis (ICA), signal space projection (SSP), canonical correlation analysis (CCA-cardiac), or denoising separation of sources (DSS-cardiac). Each algorithm is then evaluated based on its ability to remove the cardiac artefact (using the residual intensity and the improved normalised power spectrum ratio) and its ability to preserve the somatosensory evoked potentials of interest (using the signal-to-noise ratio). In a final step (right panel), the ability of canonical correlation analysis (CCA-SEP) and denoising separation of sources (DSS-SEP) to create robust somatosensory evoked potentials either with or without pre-cleaning of the cardiac artefact is tested. To achieve this aim, CCA-SEP and DSS-SEP are run on the Uncleaned data, as well as the ICA and SSP cleaned data, which were the top-performing methods from the previous stage. The performance of CCA-SEP and DSS-SEP is then quantified by means of the single-trial coefficient of variation of the somatosensory evoked potentials (which describes the variation in single-trial amplitudes across trials) and the signal-to-noise-ratio of the somatosensory evoked potentials.

Unless mentioned otherwise, all analyses were performed using Python 3.9 and MNE (https://mne.tools/stable/index.html; version 1.0.3), an open-source toolbox for analysing human neurophysiological data (Gramfort, 2013). First, to remove artefacts resulting from electrical stimulation, data were linearly interpolated between -7 and 7 ms relative to stimulus onset. Next, the data were down-sampled to 1000 Hz. The data were then notch filtered around 50 Hz with an FIR zero-phase filter and bandpass filtered between 1 and 400 Hz with a 2nd order Butterworth zero-phase filter. Data after this stage of processing are referred to as “Uncleaned” from now on. A graphical overview of the processing and analysis steps—including the employed correction methods as well as the metrics used for their evaluation—is provided in Fig. 3.

### 2.3 Cardiac artefact removal techniques

#### 2.3.1 Principal component analysis optimal basis sets (PCA-OBS)

This method is an adaptation of the method pioneered by Niazy et al. (2005) for the removal of the ballistocardiographic artefact arising from cardiac activity in simultaneous EEG-fMRI recordings. It works on each channel separately and assumes each cardiac artefact occurrence in each ESG channel is independent of all previous occurrences in time (i.e., there is no assumption of any temporal relationship between the different occurrences of the cardiac artefact in a given ESG channel). Principal component analysis is performed on a matrix of all artefact occurrences to capture the principal variations of the artefact. Then, the first *n* principal components can be selected as an optimal basis set, which can be fit to each artefact occurrence using Piecewise Cubic Hermite Interpolating Polynomials (PCHIP) and subtracted from the signal. PCA-OBS was chosen over a more conventional PCA approach to include at least one method which can be applied even in the absence of dense electrode arrays, since recording spinal responses using a limited number of electrodes has been a typical approach over many decades of ESG research as well as in recent studies (Boehme et al., 2019; Di Lionardo et al., 2021; Di Pietro et al., 2021).

While the original implementation for simultaneous EEG-fMRI is available as a plug-in for EEGLAB (https://fsl.fmrib.ox.ac.uk/eeglab/fmribplugin/), we implemented this method via custom-written scripts in Python 3.9 to use it as a correction method for ESG, which we later also contributed as a method to MNE (mne.preprocessing.apply_pca_obs). The timing of each R-peak was previously determined using automatic detection and manual correction for each participant (Nierula et al., 2024), and these latencies were used again here. A window around each R-peak was selected based on the median RR interval (the time between successive R-peaks) for each participant. It determines the time interval in which the fitted artefact is created for each heartbeat occurrence. The first four components identified by PCA as well as the mean effect were selected to form the optimal basis set—the original Niazy algorithm (Niazy et al., 2005) removes three principal components to remove the ballistocardiographic artefact, however, in our case, previous analyses (Nierula et al., 2024) revealed removing four principal components works well for cardiac artefact cleaning in our dataset. This optimal basis set is then fit to each occurrence of the artefact to form a fitted artefact, which is directly subtracted from the ESG data in each channel.

Due to the subtraction of the fitted artefact, there can be sharp edges at the beginning and end of fitting windows. To counteract this effect, the PCA-OBS algorithm was further modified here by multiplying the fitted artefact in each window with a Tukey window (α = 0.25) prior to subtraction to investigate a potential reduction of these edge effects (the results of this analysis are given in the Supplementary Material).

#### 2.3.2 Independent component analysis (ICA)

ICA is a blind source separation technique for transforming an observed multidimensional random vector into components that are statistically as independent from each other as possible (Hyvarinen, 1999). ICA was applied to the Uncleaned data using the Fast-ICA method (Hyvarinen, 1999), with the number of components specified as the number of ESG channels present across both spinal patches (the activity from both spinal patches was considered in order to maximise the information available to ICA).

The components to be removed were automatically determined in MNE, which selects components for removal based on cross-trial phase statistics. Cross-trial phase distributions are time-dependent histograms, and, in this case, these are phase histograms calculated across events, where each event is defined as a 1 second time window of the independent components, where the centre of the window is defined by the R-peak as identified from the ECG signal. Independent components associated with cardiac activity will be synchronous with the ECG trace and, therefore, exhibit a non-uniform cross-trial phase distribution (Dammers et al., 2008). The independent components are then selected for removal based on the significance value of the Kuiper statistic and the data are finally reconstructed by zeroing the excluded components and inverse transforming the data. In cortical data, this approach has been shown to be robust and highly sensitive for the removal of components attributed to cardiac activity as determined from the ECG channel (Dammers et al., 2008). Using this method, for median nerve stimulation, 13.9 ± 2.5 (mean ± std) components were removed, and 14.2 ± 3.2 components for tibial nerve stimulation.

Alternative ICA procedures were also tested: (i) applying ICA to anteriorly re-referenced data and (ii) applying ICA separately to each spinal patch (Supplementary Material).

#### 2.3.3 Signal space projection (SSP)

SSP (Uusitalo & Ilmoniemi, 1997) assumes the signals produced by different sources have different and fixed orientations in sensor space, which implies each source has a distinct and stable field pattern. Since SSP is a spatial filtering technique, the activity from both spinal patches is considered in tandem to maximise the spatial information available.

Measured signals can be divided into an estimate of the signals produced by a distinct source (here: cardiac activity) and all other sources, including spinal signals of interest. For cardiac artefact removal, SSP was performed on epochs created from 0.2 seconds before to 0.4 seconds after the identified R-peaks, thus allowing the identification of projection vectors associated with the sources of cardiac activity which can subsequently be removed.

To determine the number of projectors to remove, SSP was first performed with all number of projectors from 1 to 20 after both median and tibial nerve stimulation (20 was chosen as an upper limit, as we noticed that the signal-to-noise ratio of the SEPs of interest had decreased substantially at this point). All relevant metrics (as described later in Section 2.5) were then computed, providing residual intensity (RI; Section 2.5.1), improved normalised power spectrum ratio (INPSR; Section 2.5.2), and signal-to-noise ratio (SNR; Section 2.5.3) for cleaned data after removing different number of projectors. For the signal-to-noise ratio, the number of projectors that gives rise to the highest group-average SNR was documented. For the group-average RI and INPSR, curves were generated plotting the RI and INPSR, respectively, against the number of projectors. The number of projectors at the elbow point of these plots (representing the point of maximum curvature; Satopaa et al., 2011) was then documented in each case. An exemplary plot to illustrate this method is shown in Supplementary Figure S2. To choose the optimum number of projectors, a weighted average of the number of projectors based on the SNR, RI, and INPSR metrics was determined according to

$$\begin{array}{l} chosen\;number\;of\;projectors\\ \;\;\;\;\;\;\;\;\;\;\;\;\;\;\;\;\;\;\;\;\;\;\;\;\;\;\;\;\;\;\;\;\;\;\;\;\;\;\;\;=\;\frac{0.5\left(idx_{RI}\right)+0.5\left(idx_{INPSR}\right)+1\left(idx_{SNR}\right)}{0.5+0.5+1}, \end{array}$$

where idx represents the number of projectors recommended based on each respective metric. In all cases, the chosen number of projectors is rounded to the closest integer. We chose to implement a weighting of 0.5 for both RI and INPSR as both of these metrics evaluate characteristics related to the cardiac artefact removal performance, and we aimed to achieve an even balance between removing the artefact effectively and maintaining relevant task-evoked activity of interest (which is indicated by the SNR metric). Ultimately, five projectors were selected for removal for both median and tibial nerve stimulation (with this approach simply referred to as SSP from now on).

#### 2.3.4 Canonical correlation analysis (CCA-cardiac)

A variant of canonical correlation analysis (CCA) known as canonical correlation average regression (Waterstraat et al., 2015) was performed to find the set of weights which maximise the correlation between single-trial heartbeat events (X) and the trial-averaged heartbeat (Y) of ESG data, henceforth referred to as CCA-cardiac. CCA-cardiac was performed using the Modular EEG Toolkit (MEET; https://github.com/neurophysics/meet; Waterstraat et al., 2015) and the weights *w_x_* and *w_y_* are the weights that mutually maximise the correlation between the two inputs according to

$$\underset{w_{x}w_{y}}{\max}corr\left(w_{x}^{T}X,\;w_{y}^{T}Y\right),$$

where X and Y are both multi-channel signals. In this case, the ESG data were epoched according to the detected R-peaks for each participant—a heartbeat event is considered to be from 0.4 seconds before to 0.6 seconds after each R-peak in this case. This gives matrix X, which contains the concatenation of all heartbeat events from 1 to N for each channel [matrix size: number of channels, number of heartbeats*number of time points per heartbeat]. Matrix Y is then created, which contains the trial-averaged heartbeat event repeated N times for each channel [matrix size: number of channels, number of heartbeats*number of time points per heartbeat]. A graphical representation of the matrices relevant to CCA is shown in Supplementary Figure S3. With this construction, the weight matrix *w_x_* represents the spatial filters that maximise the correlation between the single-trial heartbeats (X) and the average heartbeat artefact (Y).

The computed weights (matrix size: number of channels, number of components; note in the case of full rank the number of components is equal to the number of channels) were then applied to the participant’s continuous raw data via multiplication (i.e., prior to epoching about heartbeat events; [matrix size: number of channels, total number of times in recording]), to form a number of components with each component having a matrix size of [1, total number of times in recording]—the same shape as previous single-channel raw data. These components are ranked according to the correlation coefficient—with component 1 representing the CCA component with the highest correlation to heartbeat activity, component 2 having a lower correlation and so on. Thus, to determine the number of components necessary for adequate removal of the cardiac artefact, the same method as used to inform the number of SSP projectors to remove was employed to determine the number of components to remove. This resulted in the removal of the top nine components for median nerve stimulation, and the top six components for tibial nerve stimulation. After the removal of the chosen number of components, the data were projected back to sensor space (Haufe et al., 2014).

#### 2.3.5 Denoising separation of sources (DSS-cardiac)

Denoising separation of sources (DSS: De Cheveigné & Parra, 2014) jointly diagonalises the covariance matrix of the original data (C0), and the covariance matrix of the data after applying a so-called bias filter that emphasises particular aspects of the signal of interest (C1). DSS was used to remove the cardiac artefact by first finding the subspace that is most repeatable across heartbeat occurrences for each participant and condition, and then projecting these subspaces out of the data. Here, the original data are the Uncleaned, raw data [matrix size: number of channels, total number of times in recording], and the biased data are the data averaged over all heartbeat events [matrix size: number of channels, number of time points per heartbeat], as described in the original publication of DSS (De Cheveigné & Parra, 2014). The trial-averaged heartbeat is the same as that previously described for CCA-cardiac, and this application of DSS is henceforth referred to as DSS-cardiac. The spatial filters obtained via DSS-cardiac can then be applied to the original raw data similarly to CCA-cardiac to obtain components [matrix size: number of components, total number of times in recording].

According to this construction and as suggested in De Cheveigné and Parra (2014), the first component provides the most repeatable linear combination of sensor signals, with each component thereafter representing an uncorrelated component with the next highest power ratio. The first x components thus provide a subspace that maximises the power of the mean cardiac signal versus total power. Therefore, to remove the cardiac artefact, the number of components to project out of the data must be determined—this was achieved using the same method that was applied to determine the number of projectors to remove for SSP and CCA-cardiac. Ultimately, nine components were projected out for the median nerve stimulation condition, and seven for the tibial nerve condition.

#### 2.3.6 Ranking of the cardiac artefact removal methods

Each artefact removal method was ranked according to their performance in terms of artefact removal (RI and INPSR) and signal preservation (SNR), in order to determine the top two methods for each condition (median or tibial nerve stimulation). For this ranking, 1 indicated the best performance, and 5 the worst. Please refer to Section 2.5 for a description of each metric, and to Table 1 for the resulting group-level RI, INPSR, and SNR for each cardiac artefact removal method. An overall score for each method was then calculated using the weighted average of the rankings, according to

**Table 1.** Group-level residual intensity (RI: smaller numbers indicate better performance), improved normalised power spectrum ratio (INPSR: larger numbers indicate better performance) and SEP signal-to-noise ratio (SNR: larger numbers indicate better performance) results for cervical and lumbar channels of interest in response to median and tibial nerve stimulation, respectively.

**Cervical Spinal Cord**			
	**RI**	**INPSR**	**SNR**
Uncleaned	-	-	2.45 ± 0.33
PCA-OBS	0.21 ± 0.04%	2.57 ± 0.06	7.95 ± 0.88
ICA	0.27 ± 0.02%	3.86 ± 0.08	11.18 ± 1.14
SSP	0.57 ± 0.06%	3.19 ± 0.08	14.37 ± 1.32
CCA-cardiac	1.24 ± 0.14%	2.57 ± 0.10	8.75 ± 0.94
DSS-cardiac	1.40 ± 0.15%	2.48 ± 0.09	8.96 ± 0.94

**Lumbar Spinal Cord**			
	**RI**	**INPSR**	**SNR**
Uncleaned	-	-	1.70 ± 0.23
PCA-OBS	0.28 ± 0.07%	2.77 ± 0.04	5.46 ± 0.61
ICA	0.10 ± 0.01%	5.03 ± 0.08	5.90 ± 0.83
SSP	0.48 ± 0.04%	4.14 ± 0.07	9.92 ± 1.24
CCA-cardiac	2.25 ± 0.28%	2.87 ± 0.08	6.35 ± 1.06
DSS-cardiac	2.29 ± 0.26%	2.69 ± 0.07	3.84 ± 0.48

The standard error of the mean (across participants) is also reported.

$$overall\;score=\;\frac{0.5\left(rank_{RI}\right)+0.5\;\left(rank_{INPSR}\right)+1\left(rank_{SNR}\right)}{0.5+0.5+1},$$

with a lower overall score indicating better performance. The RI and INPSR rankings were weighted by 0.5 in order to ensure a balance between performance in terms of the removal of the cardiac artefact, and the preservation of the neural information of interest as indicated by the SNR.

### 2.4 Signal enhancement techniques

All previous approaches (PCA-OBS, ICA, SSP, CCA-cardiac, DSS-cardiac) are specifically aimed at removing the cardiac artefact and thus are equally suitable for both resting-state and task-based recordings. However, in the context of task-based recordings as employed here, it is also possible to focus on enhancing the signal of interest, rather than removing data associated with noise. This was investigated using approaches based on both CCA and DSS. For both approaches, signal enhancement was performed on the Uncleaned data, as well as the top two methods of cardiac artefact removal for both median and tibial nerve stimulation, as determined by the ranking described above.

#### 2.4.1 Canonical correlation analysis (CCA-SEP)

CCA has been successfully applied both cortically (Stephani et al., 2020; Waterstraat et al., 2015) and spinally (Nierula et al., 2024) to enhance signals of interest. To achieve this aim here, CCA was performed similarly to the approach described for CCA-cardiac above but with some key differences. For the signal enhancement application, X contains the concatenation of all single-trial epochs from 1 to N based on the timing of median/tibial nerve stimulation for each channel [matrix size: number of channels, number of trials*number of time points per trial], and Y contains N times the average SEP for each channel [matrix size: number of channels, number of trials*number of time points per trial]. This method is referred to as CCA-SEP from here on. With this construction, the weight matrix *w_x_* represents the spatial filters that maximise the correlation between the single-trial activity (X) and the average SEP (Y). Taking into account knowledge of the expected latency of SEP’s evoked by median (13 ms) and tibial (22 ms) nerve stimulation and the limited duration of the expected response, the spatial filters *w_x_* were trained using short trial segments after stimulus delivery (median nerve stimulation: 7–37 ms, tibial nerve stimulation: 7–47 ms; these time windows were chosen in order to have ~25 ms of data after the expected spinal potential in order to have sufficient data for an accurate assessment of the noise covariance, as compared with alternative approaches which have used shorter time windows (Nierula et al., 2024)). The resulting spatial filters [matrix size: number of components, number of channels] were applied to whole length of the SEP epochs to form the components [matrix size: number of components, number of trials*number of times per trial]. A graphical representation of the matrices relevant to CCA is shown in Supplementary Figure S3. Further, for each type of peripheral nerve stimulation, only electrodes from the relevant spinal patch were included (median nerve: cervical patch; tibial nerve: lumbar patch).

We applied CCA-SEP separately in the lumbar and cervical spinal patches, for (i) Uncleaned, (ii) ICA-cleaned, and (iii) SSP-cleaned data to investigate whether CCA-SEP can provide additional benefits when combined with a dedicated artefact correction method, or whether it is sufficient by itself. The top component as ranked by the canonical correlation coefficient was selected for each participant and each method. As CCA is insensitive to the polarity of the signal, if the potential at the expected latency of the time course of the chosen component manifested as a positive peak, the data were inverted.

#### 2.4.2 Denoising separation of sources (DSS-SEP)

Denoising separation of sources for signal enhancement (DSS-SEP) was applied in a similar manner to DSS-cardiac—the only difference being that the biased data are now the data averaged over entire epochs created from all median/tibial nerve stimulation events, with an epoch starting from 0.2 seconds before stimulation and ending 0.7 seconds after stimulation (De Cheveigné & Parra, 2014). Additionally, as with CCA-SEP, for each type of peripheral nerve stimulation, only electrodes from the relevant spinal patch were included (median nerve: cervical patch; tibial nerve: lumbar patch). We thus applied DSS-SEP separately in the lumbar and cervical spinal patches, for (i) Uncleaned, (ii) ICA-cleaned, and (iii) SSP-cleaned data to investigate whether DSS-SEP can provide high-fidelity SEPs, with and without pre-cleaning of the cardiac artefact. Further, as this configuration of DSS implies the first component has the strongest possible mean effect relative to overall variability (De Cheveigné & Parra, 2014), only the first component was retained for further analysis for each participant. Similar to CCA-SEP, inversion of the component was performed if necessary.

### 2.5 Metrics

The performance of PCA-OBS, ICA, SSP, CCA-cardiac, and DSS-cardiac was quantified in two different ways. On the one hand, we assessed each method’s efficacy in terms of cardiac artefact reduction in two ways: first, in the time domain via the remaining amplitude of the cardiac artefact, referred to by the metric residual intensity (Marino et al., 2018), and second, in the frequency domain using the improved normalised power spectrum ratio of the cardiac artefact (Javed et al., 2017). On the other hand, we assessed each method’s ability to preserve or enhance the response of interest by computing the signal-to-noise ratio of the SEPs. Furthermore, the effect of CCA-SEP and DSS-SEP was examined in terms of both the signal-to-noise ratio of the SEPs, and the SEP’s amplitude variation across trials in the potential window of interest using the coefficient of variation (CoV).

All these metrics were computed at the level of individual participants; group-level results are reported as the average ± the standard error of the mean computed across participants. Apart from the metrics related to the performance of CCA-SEP and DSS-SEP (which are analysed in component space), all metrics were investigated in channels S6, SC6, and S14 for median nerve stimulation and channels S23, L1, and S31 for tibial nerve stimulation.

#### 2.5.1 Residual intensity (RI)

The data of the channels of interest were epoched from -0.3 to 0.4 seconds with respect to the detected R-peaks in order to not only include the QRS-complex, but also the P- and T-waves, and baseline corrected within a period of -0.3 to -0.2 seconds (this baseline period was chosen as it should occur before the P-wave in most participants, without risking overlap with the T-wave of the preceding heartbeat). All epochs were then averaged for each channel to form the residual evoked response. For each method (PCA-OBS, ICA, SSP, CCA-cardiac, DSS-cardiac), the residual intensity was computed for each channel by taking the root mean square (RMS) of the cleaned data and dividing it by the RMS of the Uncleaned data and multiplying by 100 to form a percentage (with smaller values indicating a better performance).

$$Residual\;Intensity=\;\frac{RMS_{cleaned}}{RMS_{uncleaned}}x\;100.$$

Last, the residual intensity computed for the three channels of interest for each electrode patch was averaged to form the final metric.

#### 2.5.2 Improved normalised power spectrum ratio (INPSR)

For each participant, the fundamental frequency of the heartbeat was computed (considered to be the average heart rate across the recording period), along with the frequency of the first four harmonics. The power spectral density of the raw data was then estimated using Welch’s method and the power in a 0.2 Hz frequency band about the fundamental frequency and the first four harmonics was subsequently extracted (Vallat & Walker, 2021; Welch, 1967) for each channel and method (PCA-OBS, ICA, SSP, CCA-cardiac, DSS-cardiac). The power within each predefined spectral range was then summed to obtain the total band power per channel. The INPSR was computed for each channel by dividing the total band power of the Uncleaned data by the total band power of the cleaned data (with larger values indicating better performance).

$$INPSR=\frac{\sum_{i=1}^{N}P_{i}^{\;\;uncleaned}}{\sum_{i=1}^{N}P_{i}^{\;\;cleaned}}$$

Last, the INPSR computed for the three channels of interest for each electrode patch was log transformed (to reduce the variability in the data as the INPSR values can span multiple orders of magnitude) and then averaged to form the final metric.

#### 2.5.3 Signal-to-noise ratio (SNR) of SEPs

The data were epoched from -0.2 to 0.7 seconds around each stimulus onset with a baseline period from -0.1 to -0.01 seconds relative to stimulus onset. These epochs were averaged across all trials to form the evoked response in each channel of interest. We then defined time windows of interest around the typical response latency (5 ms to either side of the typical N13 response and 10 ms to either side of the typical N22 response; note that the larger time window for the N22 is chosen due to the higher latency variability induced by tibial stimulation) and searched for the strongest negative deflection within these time windows. The magnitude of the largest negative deflection across the channels of interest was chosen to be the peak amplitude. The standard deviation of the baseline period was then computed for the relevant channel, and the SNR was taken to be the peak amplitude divided by the standard deviation in the baseline period.

$$\text{SNR}=\frac{Peak\;Amplitude}{Std\;Baseline\;Period}.$$

The SNR was also computed after CCA-SEP and DSS-SEP, in these cases the relevant channel refers to the chosen component.

#### 2.5.4 Coefficient of variation (CoV)

For each dataset that CCA-SEP and DSS-SEP were applied to (Uncleaned, ICA, SSP), the epochs returned after CCA-SEP or DSS-SEP were cropped according to the type of stimulation (8–18 ms for median nerve stimulation and 12–32 ms for tibial nerve stimulation). The peak-to-peak amplitude taken as the difference between the maximum negativity and the maximum positivity within each trial was then computed and the CoV was calculated based on the variation of the peak-to-peak amplitude across all trials. The CoV across trials was computed for each method both before and after the application of CCA-SEP/DSS-SEP in order to compare the impact of pre-cleaning the cardiac artefact prior to the application of CCA-SEP/DSS-SEP.

$$CoV=\;\frac{Std\left(Peak\;to\;peak\;amplitude\;across\;trials\right)}{Mean\left(Peak\;to\;peak\;amplitude\;across\;trials\right)}.$$

### 2.6 Statistics

First, considering that this dataset was acquired with relatively regular electrical stimulation (an inter-stimulus interval of 763 ms and a jitter of at most +/-50 ms), we initially assessed whether the cardiac cycle was locked to our electrical stimulation. Here, a Rayleigh test was used to determine whether or not there is significant (p < 0.05) locking of the cardiac cycle to the tibial or median nerve stimulation, using the angle from the current stimulus to the last occurring R-peak (performed using pycircstat: https://github.com/circstat/pycircstat).

Second, to assess statistical significance between the investigated artefact removal methods with respect to the calculated metrics (SNR, RI, INSPR) a 1 x 6 (SNR; levels: Uncleaned, PCA-OBS, ICA, SSP, CCA-cardiac, and DSS-cardiac) or 1 x 5 (RI and INPSR; PCA-OBS, ICA, SSP, CCA-cardiac, and DSS-cardiac) repeated-measures ANOVA was performed in each case separately for median and tibial nerve stimulation conditions. When these omnibus tests indicated significant differences, post hoc permutation tests (with the number of permutations set to 10000) in the form of one-sample, two-tailed permutation t-tests were performed exhaustively across all method comparisons. The max-T method was used to adjust the p-values to account for the number of tests within each metric, that is, controlling the family-wise error rate (Nichols & Holmes, 2002).

Finally, to assess the impact of the signal enhancement methods, we focused on two main questions:

- When a specific form of cardiac artefact removal has been applied (or not applied), should the resulting data be left unchanged, or are there additional benefits to applying signal enhancement in the form of CCA-SEP or DSS-SEP?
- When a specific enhancement method (CCA-SEP or DSS-SEP) is chosen, should the cardiac artefact be removed prior to application?

To answer the first question, 1 x 3 repeated-measures ANOVAs (levels: no enhancement, CCA-SEP enhancement, and DSS-SEP enhancement) were performed within each of Uncleaned data, ICA-cleaned data, and SSP-cleaned data, after median or tibial nerve stimulation, for both the coefficient of variation and the signal-to-noise ratio. To answer the second question, 1 x 3 repeated-measures ANOVAs (levels: Uncleaned, ICA-cleaned, and SSP-cleaned) were performed for either CCA-SEP or DSS-SEP enhancement, separately after median or tibial nerve stimulation, for both the coefficient of variation and the signal-to-noise ratio. Where significant differences were uncovered by the repeated-measures ANOVAs, post hoc non-parametric permutation t-tests were again performed as described above including correction for multiple comparisons.

## 3 Results

### 3.1 Cardiac cycle locking to stimulation

When the angle from the stimulus to the previous heartbeat is considered for all trials and participants, neither the median nerve (p = 0.55) nor the tibial nerve condition (p = 0.34) showed a significant result. When testing each participant (N = 36) and condition (median nerve, tibial nerve) separately, only 3 of the 72 conditions were significant (p_uncorrected_ < 0.05). Overall, we thus observed little evidence of locking of the cardiac cycle to the somatosensory stimulation, and no participants or conditions were thus excluded from further analysis.

### 3.2 Quantification of SEP and cardiac artefact amplitudes

While the cardiac artefact in both the cervical and lumbar spinal cord is known to be substantially larger than the SEPs of interest, to our knowledge no quantitative data exist on this yet and we thus set out to determine the respective amplitudes in our specific dataset. For this, we used the central electrode of the cervical and lumbar electrode patches (SC6 and L1, respectively) of the Uncleaned data. The average peak deflection of the cardiac artefact in the cervical spinal cord was -297 µV, while the cervical N13 response had an average peak deflection of -0.86 µV. In the lumbar spinal cord, the average peak deflection of the cardiac artefact was 657 µV, while the lumbar N22 response had an average peak deflection of -0.93 µV. When investigating this at the cortical level, we observed that for channel CP4 (target channel for median nerve stimulation), there is a peak deflection of -1.47 μV for the N20 response (first cortical response to median nerve stimulation) and 3.45 μV for the cardiac artefact, while for channel Cz (target channel for tibial nerve stimulation), the peak deflection of the P40 response (first cortical response after tibial nerve stimulation) was 8.24 versus 8.89 μV for the cardiac artefact. Thus, on average the cardiac artefact is 345 times larger than the SEPs of interest in the cervical spinal cord, and 819 times larger in the lumbar spinal cord, but just 1–2 times larger than the responses of interest at the cortical level.

### 3.3 Cardiac artefact reduction

**Fig. 4.**
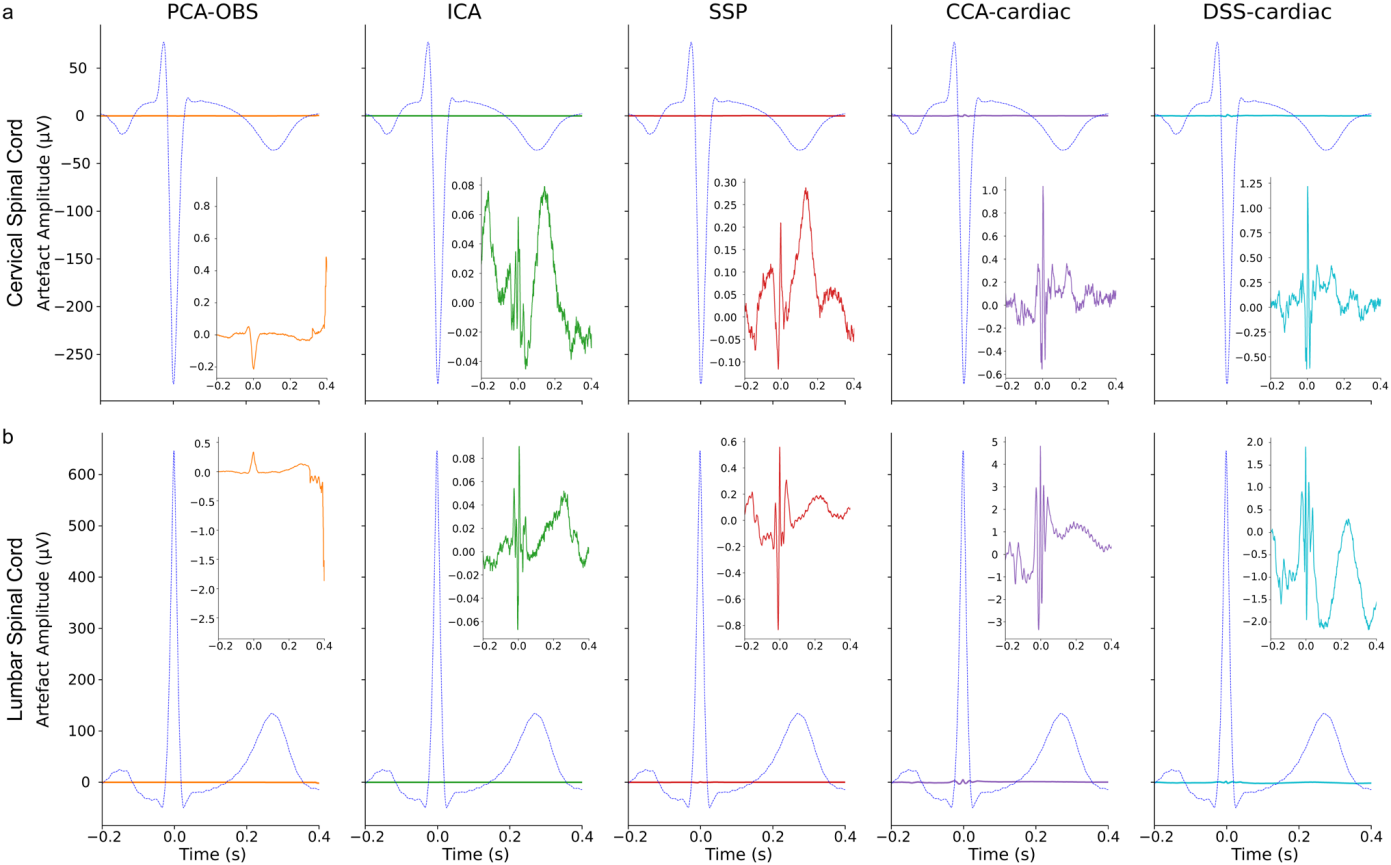
The group-average cardiac artefact in the cervical (a) and lumbar (b) spinal cord before and after cleaning. For each cardiac artefact correction method (PCA-OBS, ICA, SSP, CCA-cardiac, DSS-cardiac), we display the uncleaned artefact (blue) and the cleaned artefact (condition-specific colour) with the same y-axis scaling in the large panels. The inset panels show the cleaned artefact for each correction method (in condition-specific colour) with a y-axis scaling optimised for each method to provide as much detail as possible. Please note (i) the vastly different scale between the cleaned and uncleaned data, (ii) the different scaling between methods in the inset, and (iii) the difference of artefact amplitudes in the cervical and lumbar spinal cord. 0 seconds corresponds to the centre of the R-peak of the heartbeat.

**Fig. 5.**
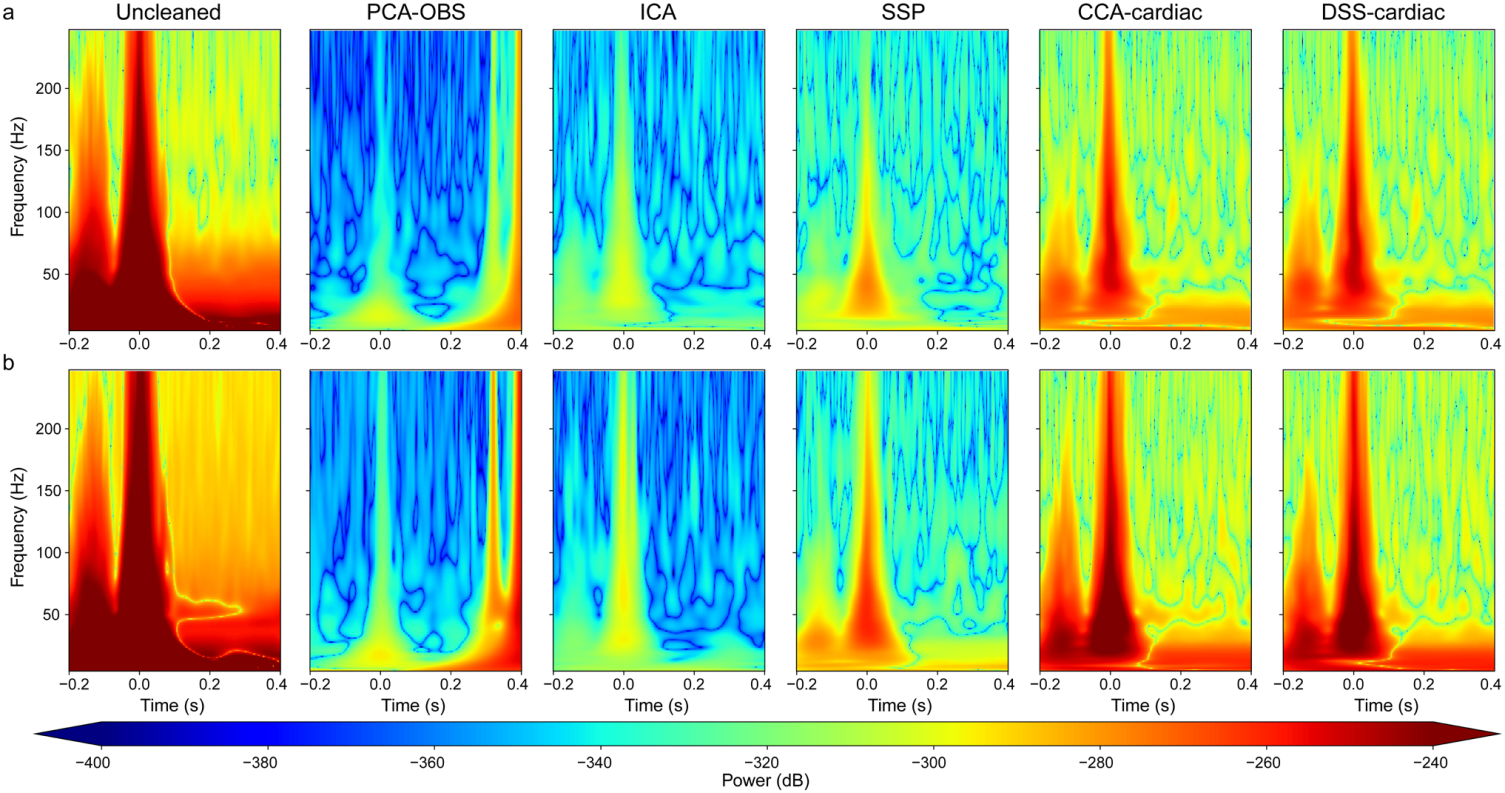
Time–frequency representations of the grand average cardiac artefact across all participants and all trials both before and after each method of cardiac artefact cleaning in (a) the cervical spinal cord (channel SC6) and (b) the lumbar spinal cord (channel L1). 0 seconds corresponds to the centre of the R-peak of the heartbeat. Note that values beyond the colour bar limits are shown as arrow heads with the same colour as indicated at the extremes of the colour bar.

First, we qualitatively investigated the reduction of the cardiac artefact via PCA-OBS, ICA, SSP, CCA-cardiac, and DSS-cardiac at the group level in the time domain (Fig. 4) as well as in the frequency domain (Fig. 5). Each method achieved a marked decrease in the amplitude and power associated with the cardiac artefact: a reduction in the peak magnitude of the R-peak in the grand-averaged waveform in cervical spinal channels from more than ~250 to ~1 µV and a reduction from more than ~600 to ~5 µV in the lumbar spinal cord in all cases. This equates to a more than ~200-fold and ~100-fold reduction in artefact amplitude at the group level for cervical and lumbar spinal cord data, respectively (exemplary single-participant time courses are shown in Supplementary Fig. S4). These time–domain results (Fig. 4) are supported by the time–frequency representations (Fig. 5), which show a severely reduced power at frequencies and time points known to correspond to cardiac activity (as shown in Fig. 1). Further, edge effects induced by PCA-OBS are visible, with sharply increasing signal amplitude observable at the border of the artefact window at 0.4 seconds (an investigation aimed at addressing these edge effects via Tukey Windows is given in the Supplementary Material).

In order to support these descriptive results, we next turned to formally assessing each method via artefact-related metrics, namely the residual intensity (RI) and the improved normalised power spectrum ratio (INPSR) of the cardiac artefact (Table 1). The RI results indicate that all methods are able to reduce the artefact to less than 1.5% at the cervical level, and less than 2.5% at the lumbar level. PCA-OBS offers the best performance at the cervical level (though this was not significantly different to ICA performance in post hoc testing; Supplementary Table S3), and ICA offers the best performance at the lumbar level (significant at the p < 0.05 level; Supplementary Table S3). The INPSR results are largely concurrent with the RI findings, though in this case, ICA offers the best performance in both the cervical and lumbar spinal cord (significant in both cases; Supplementary Table S3), both times followed by SSP. The full statistical analysis regarding methods comparison (including repeated-measures ANOVA and post hoc permutation tests) for both the median and tibial nerve conditions is given in the Supplementary Material.

### 3.4 Somatosensory evoked potentials

While the ability of each method to reduce the impact of the cardiac artefact is of central importance to this work, it is also necessary to determine whether each method can maintain meaningful neural content, that is, SEPs in our case. Thus, the impact of each method on the SEPs was visually inspected and then quantified by means of the signal-to-noise ratio (SNR) at the level of individual participants.

**Fig. 6.**
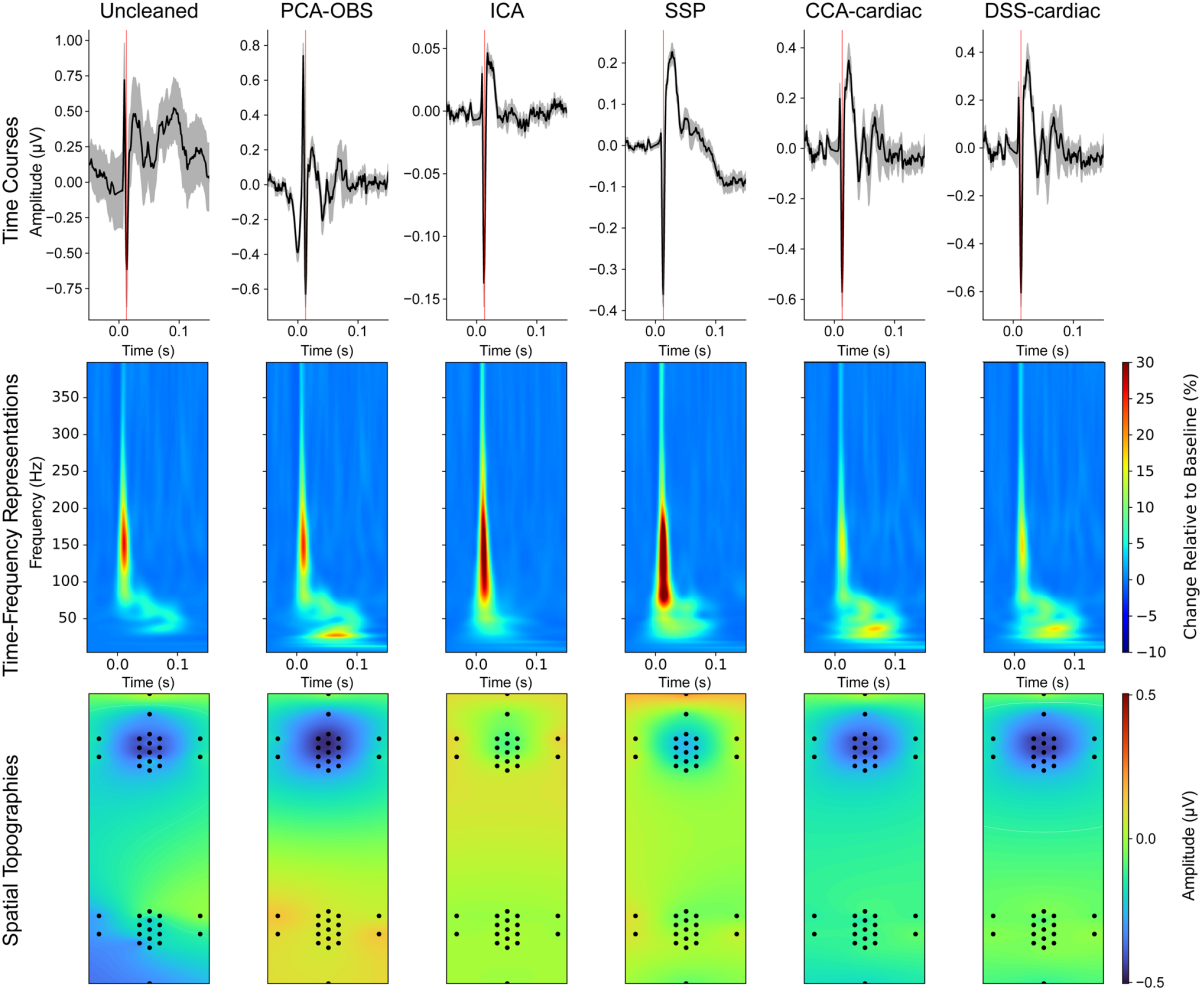
Time courses, time–frequency representations, and spatial topographies for Uncleaned, PCA-OBS, ICA, SSP, CCA-cardiac, and DSS-cardiac corrected data in response to median nerve stimulation (time courses and time–frequency representations originate from electrode SC6 in the cervical spinal cord). Note that the time courses (top) have different amplitude scales. For the time–frequency representations, the baseline period is from -0.1 to -0.01 seconds relative to stimulation. The PCA-OBS data used to generate the time–frequency representation have been linearly interpolated from -7 to 7 ms to avoid artefactual components of no interest obscuring the SEP. The expected SEP latency (13 ms) is marked by a red line in the time courses (top). The grey shading of the time courses indicates the standard error of the mean across participants. The spatial topography represents the activity from 1 ms before to 2 ms after the expected peak.

**Fig. 7.**
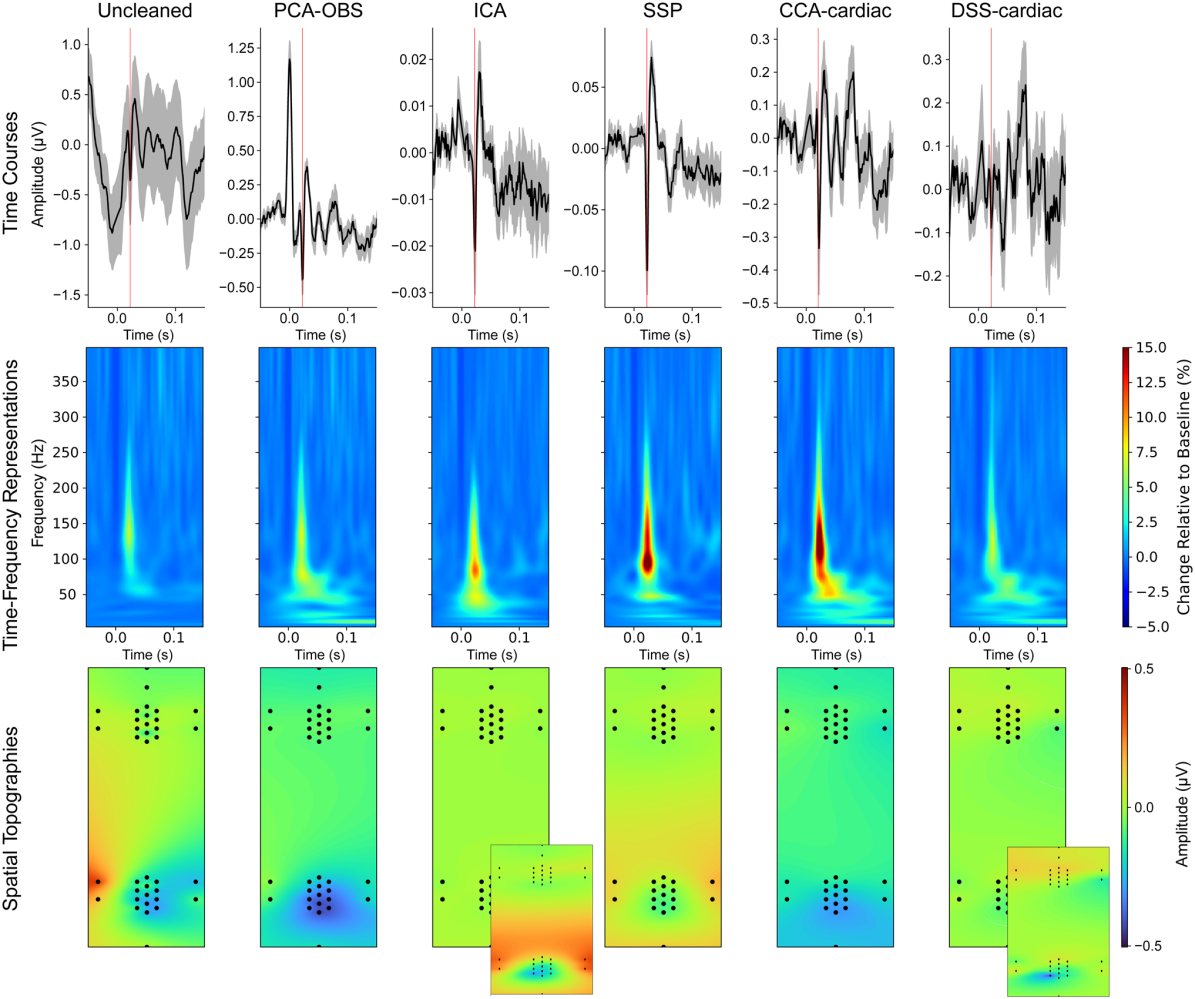
Time courses, time–frequency representations, and spatial topographies for Uncleaned, PCA-OBS, ICA, SSP, CCA-cardiac, and DSS-cardiac corrected data in response to tibial nerve stimulation (time courses and time–frequency representations originate from electrode L1 in the lumbar spinal cord). Note that the time courses (top) have different amplitude scales. For the time–frequency representations, the baseline period is from -0.1 to -0.01 seconds relative to stimulation. The PCA-OBS data used to generate the time–frequency representation have been linearly interpolated from -7 to 7 ms to avoid artefactual components of no interest obscuring the SEP. The expected SEP latency (22 ms) is marked by a red line in the time courses (top). The grey shading of the time courses indicate the standard error of the mean across participants. The spatial topography represents the activity from 1 ms before the expected peak to 2 ms after. Channel S34 has been excluded as a bad channel for participant 34. For clearer visualisation, a differently scaled spatial topography has been included (inset) for ICA and DSS-cardiac, with the colours ranging from -0.2 to 0.2 μV.

In all instances, with the exception of DSS-cardiac for tibial nerve stimulation, by removing the cardiac artefact it is possible to reveal visually distinct somatosensory evoked potentials (Fig. 6: top row, expected N13 potential latency marked in red; Fig. 7: top row, expected N22 potential latency marked in red; note the different scaling for the time courses in both figures); exemplary single-participant time courses are shown in Supplementary Figure S5. This is further supported by the time–frequency representations (Fig. 6: cervical spinal cord; Fig. 7: lumbar spinal cord; middle row) which show clear increases in power above baseline levels at the expected latency of the SEP (median nerve stimulation: 13 ms, tibial nerve stimulation: 22 ms) in all cases. Finally, the robustness of each method can be examined by its ability to localise the spinal activity to the correct electrode patch (cervical spinal cord for median nerve stimulation (Fig. 6: bottom row) and lumbar spinal cord for tibial nerve stimulation (Fig. 7: bottom row).

**Fig. 8.**
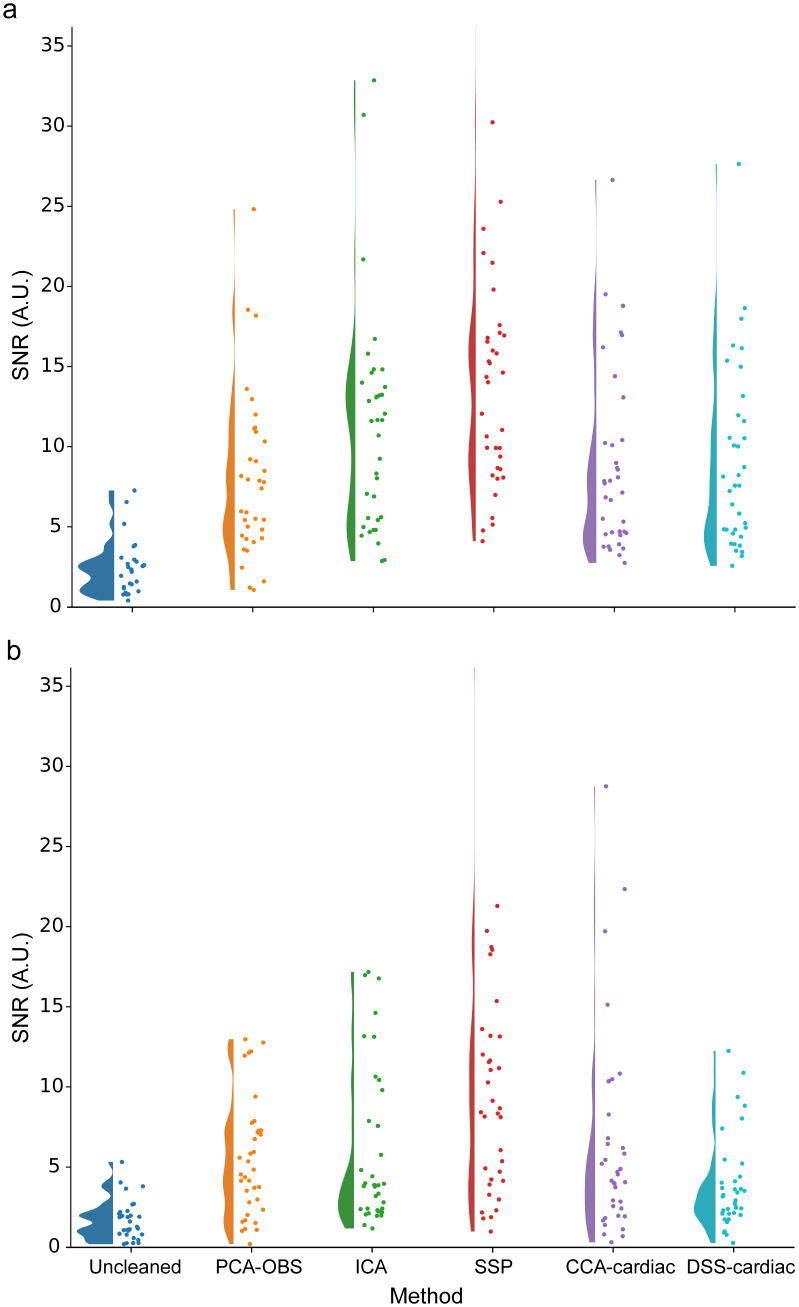
SEP signal-to-noise ratio (SNR) results for Uncleaned data and each method of cardiac artefact removal in (a) the cervical spinal cord and (b) the lumbar spinal cord, with circles indicating participants. For SSP, in the cervical spinal cord, participant 36 is not shown (SNR = 43.57) and in the lumbar spinal cord, participant 31 is not shown (SNR = 37.83).

Overall, all methods show a marked improvement in the SNR of the signal of interest (Table 1; Fig. 8) versus the Uncleaned data. Here, SSP exhibits by far the best performance in both the cervical and lumbar spinal cord, with a more than fivefold increase in SNR (statistically supported by repeated-measures ANOVA and post hoc testing; Supplementary Table S3).

### 3.5 Overall ranking of the cardiac artefact removal methods

To determine the top two performing cardiac artefact removal methods, each method was ranked in terms of its ability to both remove the cardiac artefact (RI and INPSR) and maintain the relevant spinal signals (SNR) for each condition (median or tibial nerve stimulation). When these rankings were combined into an overall score via a weighted average, ICA and SSP were the best performing methods in both the cervical and lumbar spinal cord (Supplementary Table S4).

### 3.6 Signal enhancement

Finally, we also approached this topic from a signal enhancement perspective. Signal enhancement via CCA-SEP and DSS-SEP was applied to both the Uncleaned data and data pre-cleaned using the top two performing cardiac artefact removal methods (ICA-cleaned and SSP-cleaned data). We wanted to determine whether (i) when a specific form of cardiac artefact removal is chosen (or no cleaning is performed), are there additional benefits to signal enhancement and whether (ii) when a specific form of signal enhancement is chosen, should targeted cleaning of the cardiac artefact be performed prior to this enhancement. As outcome metrics, we employed the signal-to-noise ratio of the SEP response as well as the coefficient of variation (CoV), which allowed us to additionally determine the sensitivity to detecting SEPs at the single-trial level.

**Fig. 9.**
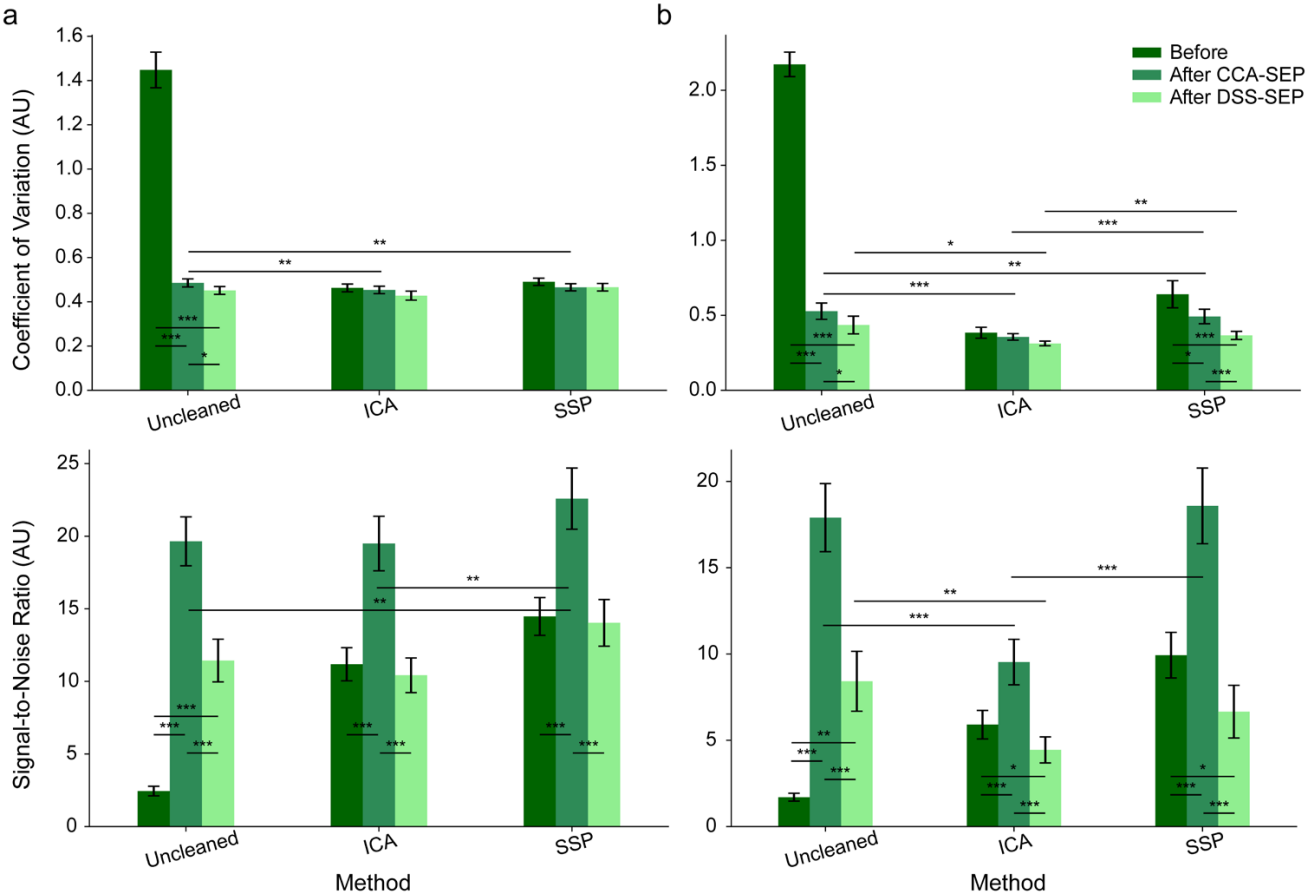
Coefficient of variation (top row) and signal-to-noise ratio results (bottom row) before and after the application of CCA-SEP and DSS-SEP in (a) the cervical spinal cord and (b) the lumbar spinal cord. Smaller CoV numbers indicate lower variation across trials, while higher SNR values indicate clearer SEPs. Error bars indicate the standard error of the mean computed across participants. Asterisks show significant comparisons after one-sample permutation t-tests (*: p < 0.05, **: p < 0.01, ***: p < 0.001).

Overall, both CCA-SEP and DSS-SEP are seen to significantly reduce the single-trial variation and increase the signal-to-noise ratio when applied directly to Uncleaned data, with CoVs 3–4 times lower and SNRs 5–10 times higher (Fig. 9; Supplementary Table S5). For CCA-SEP, pre-cleaning with ICA and SSP yields significantly lower CoVs in the cervical and lumbar spinal cord as compared with direct application on Uncleaned data, with ICA-cleaned data yielding the lowest CoVs in both cases. For DSS-SEP in the cervical spinal cord, there are no significant differences between Uncleaned and pre-cleaned data, though in the lumbar spinal cord, both ICA and SSP pre-cleaned data yield significantly lower CoVs, with ICA again performing significantly better than SSP. Only for SSP in the lumbar cord is there a significant benefit for CoV reduction when additionally employing CCA-SEP and DSS-SEP.

In all cases, CCA-SEP leads to significantly higher SNRs compared both with no enhancement and DSS-SEP enhancement. In both the cervical and lumbar spinal cord, the highest SNRs are observed using the combination of SSP and CCA-SEP. Additionally in the lumbar spinal cord, applying DSS-SEP can actually significantly lower SNRs in data pre-cleaned with ICA or SSP, though DSS-SEP can still offer benefits with direct application on Uncleaned data in both cervical and lumbar data.

For full reporting on the relevant statistics, see the Supplementary Table S6. Grand-average SEP time courses, as well as exemplary single-trial depictions after the application of CCA-SEP and DSS-SEP, are also shown in the Supplementary Material (Supplementary Figs. S6–S8).

## 4 Discussion

In this study, we systematically examined the potential of several algorithms to reduce the impact of physiological noise of cardiac origin in spinal cord electrophysiology data. Removing cardiac artefacts is of utmost importance in ESG data, considering that they are between two and three orders of magnitude larger than the evoked potentials of interest. We investigated the impact of several common artefact correction methods on both the cardiac artefact and the somatosensory evoked potentials of interest. In addition, we also tested the effects of two different signal enhancement methods, both in isolation and in combination with artefact correction methods. Together, these analyses allowed us to determine and compare the strengths and weaknesses of each method in the spinal domain.

### 4.1 Overview of each artefact removal method’s performance

Our investigations suggest that each method can effectively reduce the impact of the cardiac artefact as compared with uncleaned ESG data—with ICA followed by SSP performing best in both the cervical and lumbar spinal cord in their ability to preserve neural information of interest while removing unwanted noise.

#### 4.1.1 PCA-OBS

The application of PCA-OBS (Niazy et al., 2005; Nierula et al., 2024) more than doubled the SNR of spinal SEPs as compared with Uncleaned data in both the cervical and lumbar spinal cord and was also seen to significantly reduce the impact of the cardiac artefact, for example, showing a residual artefact intensity of just below half a percent. Despite this, the PCA-OBS algorithm can result in sharp voltage deviations at the beginning and end of fitting windows around each heartbeat. While this is not directly of concern when studying evoked responses, it presents an inherent disadvantage when it comes to resting-state recordings (Wang et al., 2021). Thus, we present an updated algorithm which includes the multiplication by a Tukey window to smooth these so-called edge effects and thereby enable the use of the PCA-OBS algorithm in cases where edge effects present problems for analysis. While this can have a slight negative effect on the ability to effectively remove the influence of cardiac activity, it still represents a viable avenue to consider for future research. Additionally, a prominence around the time of electrical stimulation (0 seconds) can be observed due to an interaction between interpolation around stimulus onset (to remove the stimulus artefact) and the PCA-OBS fitting algorithm. While this effect can be strong in a few participants, it is not a consistent effect across participants and trials and more importantly has no bearing on the results presented here as the effect is localised to the interpolation zone which is not analysed.

#### 4.1.2 SSP

The use of SSP (Uusitalo & Ilmoniemi, 1997) to remove the cardiac artefact was most successful for our dataset in terms of the SEPs of interest. SSP achieved the highest SNR of the methods tested in both the cervical and lumbar spinal cord, with a more than fivefold increase compared with Uncleaned data, alongside effective removal of the cardiac artefact as evidenced, for example, by the residual artefact intensity of ~0.5%. While the number of projectors selected for removal is relatively high (five in our case), this is warranted due to the complex spatial and temporal pattern elicited by cardiac activity (Singh & Krishnan, 2023; Taccardi, 1963). It is also not unexpected to remove a relatively high number of dimensions for complex artefacts: for example, in EEG recordings contaminated by transcranial magnetic stimulation (TMS), six to nine projectors are deemed necessary for successful artefact removal (Mutanen et al., 2016). To avoid unnecessarily removing neural content of interest, the number of projectors selected for removal should be optimised for each individual study, as we have done here. Additionally, SSP can offer further advantages over the PCA-OBS approach, as noise not related to cardiac activity but located in the artefact subspace can also be effectively attenuated by SSP (Vosskuhl et al., 2020). Despite the overall excellent performance of SSP in eliciting high-fidelity SEPs, the unexpected absence of the P11 potential from the cervical SEP time course after SSP cleaning is worthy of mention, as this is a well-documented pre-synaptic spinal potential after median nerve stimulation (Desmedt & Cheron, 1982).

#### 4.1.3 ICA

ICA (Hyvarinen, 1999) has been successfully applied in invasive spinal electrophysiology recordings to remove cardiac interference (Wang et al., 2021), and in surface electromyography of back muscles (Jiang et al., 2021), and was the top-performing method in this study with the best overall score in both the cervical and lumbar spinal cord. In our investigations, ICA was capable of both reducing the impact of the cardiac artefact and retaining spinal content of interest (as evidenced by the higher SNR as compared with Uncleaned data). Despite this, ICA did offer lower SNRs versus SSP at the cervical and lumber spinal level, which may indicate over-removal of the cardiac artefact due to the relatively high number of components removed. Such an effect has also been noted in studies attempting to remove the ballistocardiographic artefact in simultaneous EEG-fMRI (Bullock et al., 2021). Further studies may thus optimise an ICA approach by (i) finding the critical number of components to remove that better balances cardiac artefact removal and signal preservation or (ii) considering components to remove based on the cardiac activity detected in lateral electrodes along the surface of the back, which should capture the variance of the heartbeat in a more similar capacity to the spinal electrodes of interest, as opposed to the ECG channel itself—though this remains beyond the scope of this work.

#### 4.1.4 CCA-cardiac

Variations of CCA-cardiac have been used in both ESG (Mehra et al., 2025) and simultaneous EEG-fMRI (Assecondi et al., 2009) in previous research. In our study, despite offering strong performance in terms of the SNR of the evoked response, the cardiac artefact removal performance was less satisfactory with a residual intensity above 1% in both the cervical and lumbar spinal cord regions, and among the worst INPSRs of the methods included here. Further, after cleaning tibial nerve stimulation data with CCA-cardiac, the expected spatial pattern of the N22 response—a response localised in the centre of the lumbar spinal cord patch—is not evident, indicating a drop in spatial specificity. Although alternative methods were more successful than CCA-cardiac in our study, when higher-dimensional ECG recordings are performed (as opposed to a single ECG electrode in this dataset), the selection of components to remove can be achieved by identifying components in the ESG data that are most correlated to the ECG leads (in contrast to the correlation with the average across all heartbeat events in the spinal data itself as performed here), which may lead to improved performance (Mehra et al., 2025).

#### 4.1.5 DSS-cardiac

Though DSS-cardiac has been suggested for the removal of cardiac artefacts in MEG data (De Cheveigné & Parra, 2014), it was found to be least suitable for ESG data in the present study. This method did not sufficiently remove the cardiac artefact—as evidenced by the highest RI and lowest INPSR of the methods examined here. Despite offering higher SNRs for the SEPs of interest versus Uncleaned data, DSS-cardiac ranked lowest among the cardiac artefact removal techniques under study. DSS-cardiac was implemented as suggested by De Cheveigné and Parra (2014), using the average across heartbeat events as the biased data to identify a subspace that maximises the power of the mean cardiac signal versus the total power, which could then be projected out of the data. As such, no additional spectral filtering was performed. However, given the cardiac artefact is most dominant at frequencies below ~30 Hz, it is possible that additionally biasing the data by low pass filtering at 30 Hz could lead to the identification of a more relevant artefact subspace for removal.

### 4.2 Overview of signal enhancement performance

As an alternative approach to the cardiac artefact removal methods, we assessed the performance of methods for signal enhancement by leveraging variants of CCA and DSS. CCA-SEP, in particular, clearly enhanced the SEPs of interest, providing robust responses even at the single-trial level, and was shown to be relatively insensitive to pre-cleaning of the cardiac artefact, though a combination with SSP or ICA can lead to further improvement. In terms of the signal-to-noise ratio, the combination of SSP and CCA-SEP was more successful than a combination with ICA. This could be related to the removal of just 5 spatial projections for cardiac artefact cleaning with SSP versus approximately 14 components for ICA, thus leaving enough relevant neural information in the case of SSP prior to signal enhancement.

CCA has previously been shown to be effective for spinal recordings (Nierula et al., 2024), though this is the first time that this method has been shown to function well in spinal recordings even in the absence of dedicated cleaning of the cardiac artefact. While DSS-SEP has been applied successfully in MEG research (De Cheveigné & Parra, 2014; De Cheveigné & Simon, 2008), it only led to increased SEP SNRs in this ESG dataset when applied to Uncleaned data, and was not found suitable for use in conjunction with prior cleaning of the cardiac artefact. Further, the SEP SNR was lower in all cases as compared with CCA-SEP, despite offering lower single-trial variability. This difference in performance between CCA-SEP and DSS-SEP could be driven by the fact that in this study, for CCA-SEP the algorithm was trained on short ~35 ms time windows about stimulation triggers, whereas DSS-SEP was trained on entire epochs (-0.2 to 0.7 seconds relative to stimulation) as suggested by De Cheveigné and Parra (2014). This could have provided an advantage for CCA-SEP, allowing for the generation of spatial filters that are more specific to the stimulus-evoked activity. Overall, however, both CCA-SEP and DSS-SEP can provide effective signal enhancement even in the absence of prior cardiac artefact removal.

In this study, we selected solely the top component as ranked by the correlation coefficient (CCA-SEP), or power ratio (DSS-SEP). However, some studies have demonstrated that (i) the “best” component may not always be represented by the first component of CCA (Nierula et al., 2024; Stephani et al., 2021) and that (ii) a combination of multiple components may be used for signal enhancement for both CCA and DSS approaches—as has been performed in previous research (De Cheveigné & Parra, 2014; De Cheveigné et al., 2018) and was performed in this study for cardiac artefact cleaning. Further, for CCA-SEP, previous studies have also included the spatial pattern (computed by multiplying the covariance of the data by the spatial filters (Haufe et al., 2014)) as a decision criterion for selecting the “optimal” component, in the sense of the “spatio-temporal component of most interest” (Nierula et al., 2024; Stephani et al., 2021). Allowing for additional flexibility in component selection may, therefore, enable better enhancement performance via both CCA and DSS in future studies.

### 4.3 Method selection depends on recording setup and research aims

For removal of the cardiac artefact, in instances where electrode arrays are extensive enough to make use of spatial filtering techniques, both ICA and SSP (with the number of projectors optimised for each individual study) offer excellent results. However, due to the construction and subsequent removal of artefact subspaces via the spatial filtering approaches considered here (ICA, SSP, CCA-cardiac and DSS-cardiac), it is possible that these methods might remove relevant neural information alongside unwanted noise. This can result in smaller SEP peak deflections—though higher SNRs compared with uncleaned data, as seen in this study, indicate these methods are overall effective at balancing the removal of unwanted noise with the preservation of neural content of interest. This effect is not uncommon, and has been observed in other studies (for an example in relation to SSP, see Haumann et al., 2016). In cases where small number of electrodes (or even single electrodes) are used to record spinal signals and techniques which rely on multi-channel recordings cannot be utilised, PCA-OBS offers sufficient performance. As the standard implementation of PCA-OBS can result in the introduction of sharp voltage deviations, its use may be problematic in the case of resting-state recordings in the spinal cord (though Tukey windows can aid in reducing this effect). Further improvements may be offered in the cervical cord via the use of anterior re-referencing (Nierula et al., 2024), though this was not implemented here. Based on our analyses, CCA-cardiac and DSS-cardiac offer lower overall performance versus the alternative cardiac artefact removal techniques explored here.

Additionally, in cases of task-based recordings with multiple electrodes and no necessity to analyse raw data traces, it is possible to use CCA-SEP to obtain high-fidelity somatosensory evoked potentials, as demonstrated here. This can be pursued without targeted cleaning of the cardiac artefact (as CCA-SEP’s performance was sufficient alone), though a combination with SSP can lead to further sensitivity gains. Leveraging spatial filtering approaches like CCA-SEP can enable the extraction of more robust spinal SEPs and may be particularly useful in instances where large trial counts are unsuitable, for example, in pain research. Here, it was also possible to obtain clear somatosensory evoked potentials at the single-trial level, which opens further paths for analysis, including the investigation of the fluctuation of response amplitudes across different processing levels of the peripheral and central nervous system (Nierula et al., 2024). DSS-SEP was unable to offer the same benefits as CCA-SEP in terms of the overall SNR of the SEPs of interest with the setup employed in this study. Importantly, it must be noted that both CCA-SEP and DSS-SEP in the configuration applied here rely on there being a clear mean effect, which may not be the case in low signal-to-noise ratio situations—in such a case DSS-SEP offers a distinct advantage as it is possible to choose alternative bias filters to circumvent this concern.

### 4.4 Advantages of cardiac artefact denoising over classical approaches

Beyond enabling single-trial investigations, the cardiac denoising methods included in this investigation have significant advantages over classical approaches to limit the impact of the cardiac artefact. As mentioned previously, these approaches include (i) cardiac gated stimulation, (ii) trial averaging, and (iii) high-pass filtering. Cardiac-gated stimulation involves stimulating only during artefact-free periods of the cardiac cycle (Cracco, 1973), and while it is an effective means to eliminate the cardiac artefact, it does not allow for studies where cardiac-somatosensory interactions are of interest and stimulation must be distributed across the cardiac cycle (Al et al., 2020). In contrast, the noise reduction methods employed in this investigation do not preclude examining such interactions. Further, for successful trial averaging, typically ~2000 trials are necessary (Cruccu et al., 2008). However, modern cognitive neuroscience paradigms often consist of conditions with low number of trials (e.g., deviance detection designs in the context of predictive processing (Tabas et al., 2020)), therefore, the use of cardiac denoising methods that provide high-fidelity signals even with low trial counts, such as exhibited here, is vital. Finally, ESG studies often filter out content below 30 Hz (Lastimosa et al., 1982) which helps to remove frequency content associated with cardiac activity. While high-pass filtering is not problematic in the investigation of spinal somatosensory evoked potentials, it is undesirable in the case of resting-state recordings, where frequencies of interest can lie below 30 Hz (Wang et al., 2021). The methods shown here are proven effective even without such extensive high-pass filtering, and are thus more suitable for studies involving resting-state recordings.

### 4.5 Alternative approaches

While this study did not exhaustively examine all possible methods to reduce the impact of the cardiac artefact in spinal electrophysiology recordings, to the best of our knowledge, it represents the first study to evaluate how well common methods for artefact removal perform in the spinal domain. Alternative approaches not considered in the present study—but which have had varying success in cardiac artefact removal in alternative domains—include, for example, template-based subtraction (Chander et al., 2022; Stenner et al., 2025), adaptive template subtraction (Gracia et al., 2024), empirical mode decomposition in combination with principal component analysis (Javed et al., 2017), deep learning (McIntosh et al., 2021), and harmonic regression approaches (Krishnaswamy et al., 2016). Additionally, given the promising results of ICA in terms of cardiac artefact removal, and the ability of SSP to improve the SNR of our signal of interest, it may be possible in the future to create a hybrid method that balances the advantages of each approach and can more fully remove the impact of the cardiac artefact without compromising the quality of the signal of interest (this has been attempted with some success with respect to blink correction in EEG recordings (Korpela et al., 2012)). Recent attempts have also been made to avoid the need for artefact removal altogether by instead excluding all trials contaminated by a cardiac event (Gabrieli et al., 2025). While this resulted in high-quality data and is thus a viable approach, it led to exclusion of ~30% of all trials, which is not possible, for example, in paradigms with low trial counts and precludes the investigation of the interaction between stimulation and cardiac events or the analysis of continuous resting-state signals.

Ultimately, the difficulty in removing the cardiac artefact from spinal electrophysiology recordings lies in its large amplitude relative to the spinal activity of interest, and the spatial and temporal non-stationary characteristics of cardiac activity (i.e., there can be changes in the timing and shape of heartbeat occurrences over time (Appel et al., 1989; Huck et al., 2000)). As all methods tested here assume temporal stationarity, to achieve near complete removal of the cardiac artefact, it may become necessary to look beyond current methods and develop improved approaches tailored to the specific and dynamic activity of the heart.

### 4.6 Limitations

There are several limitations in the present study worthy of mention. First, while there was no significant locking of the cardiac cycle to the somatosensory stimulation, in cases of such temporal locking, the cardiac artefact removal methods examined here may perform sub-optimally. Second, while this study presents an analysis of several common algorithms, it is by no means an exhaustive study of all possible cardiac artefact removal methods and thus there might exist even better methods to remove the cardiac artefact in spinal electrophysiological recordings. Third, due to the sharp and transient nature of the high-amplitude R-peak in ESG data, the application of filtering prior to the removal of the cardiac artefact may introduce filter ringing. Thus, the type and order of filters should be carefully considered, as well as the order of application in data pre-processing stages. Specifically, notch filtering might be best performed after cardiac artefact correction. Finally, since the results of this study are specific to our high-density recording montage and may vary in alternative recording setups, it is important to consider the specifics of each study in order to select the appropriate algorithm for each individual use case.

### 4.7 Conclusion

Experimental research in non-invasive electrophysiology of the human spinal cord is currently only being performed by few research groups and there has thus far been no focus on determining effective methods to reduce the effect of the cardiac artefact. This study evaluated some of the most common artefact reduction techniques in imaging neuroscience and examined their efficacy at removing the cardiac artefact in electrospinography data. When electrode arrays are extensive enough to allow for spatial filtering, SSP and ICA perform well, while in cases where the recording montage does not permit this, PCA-OBS and its variations should be considered depending on the aims of study. Further, using CCA allows for obtaining high-fidelity somatosensory evoked potentials, even in the absence of dedicated cleaning methods. Taken together, our investigations provide viable approaches for increasing the robustness and sensitivity of electrospinography across different recording setups as well as different experimental scenarios, such as task-based or resting-state investigations.

## Supplementary Material

Supplementary Material

## Data Availability

The underlying data and code are openly available via OpenNeuro (https://openneuro.org/datasets/ds004388) and GitHub (https://github.com/eippertlab/cardiac-artefact-removal), respectively.
