# Supplementary material for: Evaluating cardiac noise correction approaches for non-invasive electrophysiology of the human spinal cord
Source: Imaging Neurosci (Camb). 2025 Oct 22;3:IMAG.a.938. doi: 10.1162/IMAG.a.938 (PMC12547440; doi:10.1162/IMAG.a.938)
Supplement: Supplementary Material [file IMAG.a.938_supp.pdf]

Supplementary Material

Evaluating cardiac noise correction approaches for non-invasive electrophysiology of the human spinal cord

Emma Bailey[1*+], Birgit Nierula[1a*], Tilman Stephani[2b], Burkhard Maess[3], Vadim Nikulin[2], Falk Eippert[1+]

[1] Max Planck Research Group Pain Perception, Max Planck Institute for Human Cognitive and Brain Sciences, Leipzig, Germany

[2] Research Group Neural Interactions and Dynamics, Department of Neurology, Max Planck Institute for Human Cognitive and Brain Sciences, Leipzig, Germany

[3] Methods and Development Group Brain Networks, Max Planck Institute for Human Cognitive and Brain Sciences, Leipzig, Germany

[*] These authors contributed equally

[+] Corresponding authors

[a] Current address: Fraunhofer Heinrich-Hertz-Institute, Department for Vision and Imaging Technology, Interactive and Cognitive Systems Group, Berlin, Germany

[b] Current address: Donders Institute for Brain, Cognition and Behaviour, Radboud University, Nijmegen, the Netherlands

Address for correspondence:

Emma Bailey & Falk Eippert

Max Planck Research Group Pain Perception

Max Planck Institute for Human Cognitive and Brain Sciences

Stephanstraße 1a, 04103, Leipzig, Germany

Phone: +49 341 9940 2673

bailey@cbs.mpg.de

eippert@cbs.mpg.de

1. The effect of a Tukey window within the PCA-OBS algorithm

As noted in the main manuscript, the PCA-OBS algorithm can introduce sharp voltage deviations at the beginning or end of artefact fitting windows, which is particularly detrimental in the case of resting state recordings. To address this, the ability of a Tukey window to reduce this effect was first examined visually by probing the ability to reduce edge effects apparent in the raw data traces. Figure S1 demonstrates the effect of a Tukey window on the raw data traces of a single participant (sub-020), where the original PCA-OBS algorithm is seen to leave sharp deviations in voltage at the edges of fitting windows of the artefact. The updated algorithm that includes the multiplication by a Tukey window is able to effectively smooth these rifts, though it does introduce low-frequency valleys in their place.

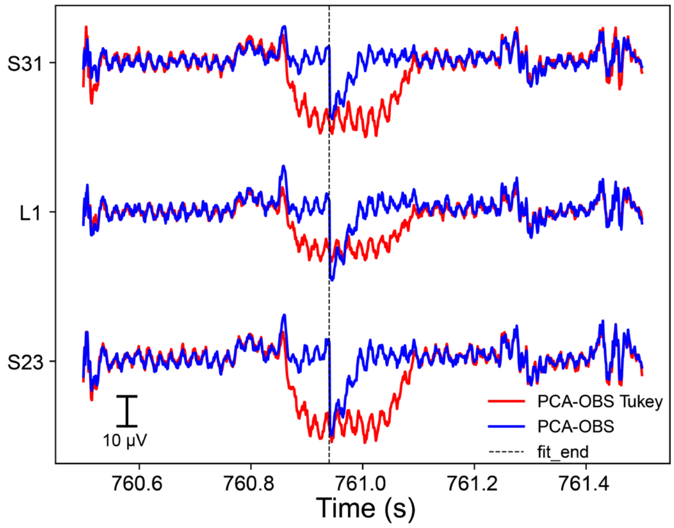

Figure S1: The effect of the Tukey window (red) on raw data traces from 3 lumbar spinal channels demonstrating the effect in a single participant (sub-020) alongside the original PCA-OBS algorithm (blue); fit_end marks the end of a fitting window about a heartbeat occurrence.

The effect of the Tukey window modification as compared to the original PCA-OBS algorithm was then examined at the group-level, by computing the relevant metrics, as seen in Table S1. The results reveal that INPSR and RI (i.e. the ability to effectively remove the cardiac artefact) are negatively affected by the application of a Tukey window. However, the effect on the somatosensory evoked potentials of interest, as seen in the SNR, is negligible. Given the mixed results when applying a Tukey window, choosing whether or not to implement this additional step will depend on the specific aims of the research and how important it is to avoid the sharp voltage deviations introduced by PCA-OBS.

Table S1: The results in terms of residual intensity (RI), improved normalised power spectrum ratio (INPSR) of the cardiac artefact, and the signal-to-noise ratio (SNR) of the SEPs for both median and tibial nerve stimulation, calculated across all participants, for the original PCA-OBS algorithm, and the PCA-OBS algorithm including a Tukey window.

	**RI**		**INPSR**		**SNR**	
	Median	Tibial	Median	Tibial	Median	Tibial
**PCA-OBS**	0.21%	0.28%	2.57	2.77	7.95	5.46
**PCA-OBS TUKEY**	1.88%	2.76%	1.70	1.58	7.53	5.40

2. Alternative ICA approaches

As ICA was one of two top-performing cardiac artefact removal methods in this study, but with a consistently lower signal-to-noise ratio for the SEPs of interest as compared to SSP, the ability of ICA to effectively remove the cardiac artefact while retaining spinal signals of interest was further investigated to determine whether alternative configurations could yield better results. In the context of this study, the ICA pipeline was altered to determine if ICA could perform better i) with anteriorly re-referenced data (ICA-Anterior), or ii) when applied separately to the cervical and lumbar spinal patches (ICA-Separated). As the results in Table S2 demonstrate, there are no consistent advantages for either ICA alternative tested (with ICA-Separated performing worst).

For ICA-Anterior, 13.8 ± 2.4 components were removed in the median nerve condition, and 14.1 ± 3.3 components in the tibial nerve condition. For ICA-Separated, 4.8 ± 1.8 and 11.1 ± 2.5 components were removed for the median and tibial nerve stimulation conditions, respectively.

Table S2: Resulting residual intensity (RI), improved normalised power spectrum ratio (INPSR) of the cardiac artefact, and the signal-to-noise ratio (SNR) of the SEPs of the alternative configurations of ICA tested, computed across all participants.

	RI	***INPSR***	***SNR***
		**Cervical Spinal Cord**	
ICA	0.27%	3.86	11.18
ICA-Anterior	0.30%	3.77	11.63
ICA-Separated	2.31%	2.48	7.78
		**Lumbar Spinal Cord**	
ICA	0.10%	5.03	5.90
ICA-Anterior	0.12%	4.95	5.83
ICA-Separated	2.60%	3.10	4.37

3. Approach for choosing the number of projectors/components for SSP, CCA-cardiac and DSS-cardiac

To determine the number of projectors (SSP) or components (CCA-cardiac and DSS-cardiac) required to remove the cardiac artefact while still preserving spinal signals of interest, three separate result curves were generated for each method (SSP, CCA-cardiac, DSS-cardiac) and condition (median or tibial nerve stimulation), which show the group-average i) SNR, ii) RI and iii) INPSR against the number of projectors/components. An example can be seen in Figure S2 for CCA-cardiac in the cervical spinal cord after median nerve stimulation. The number of projectors/components removed that leads to the highest SNR, as well as the number of projectors/components at the elbow point of the RI and INPSR plots were documented in each case. The number of projectors/components to remove was then selected based on the weighted average of these three values, as described in the main manuscript.

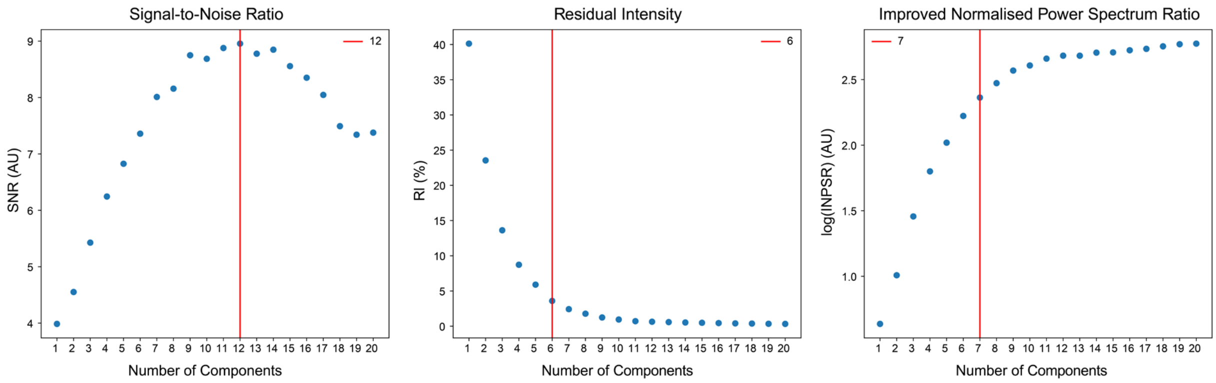

Figure S2: The chosen number of projectors (red line) based on the highest signal-to-noise ratio (left) as well as the elbow point of the residual intensity (middle) and improved normalised power spectrum ratio (right) for median nerve stimulation in the cervical spinal cord after the application of CCA-cardiac.

4. Matrix inputs and outputs for CCA

To provide more insights into how CCA is used in this study, Figure S3 depicts the way the inputs are created for CCA (a-c) and the way in which the output from CCA is used to generate component time-courses (d).

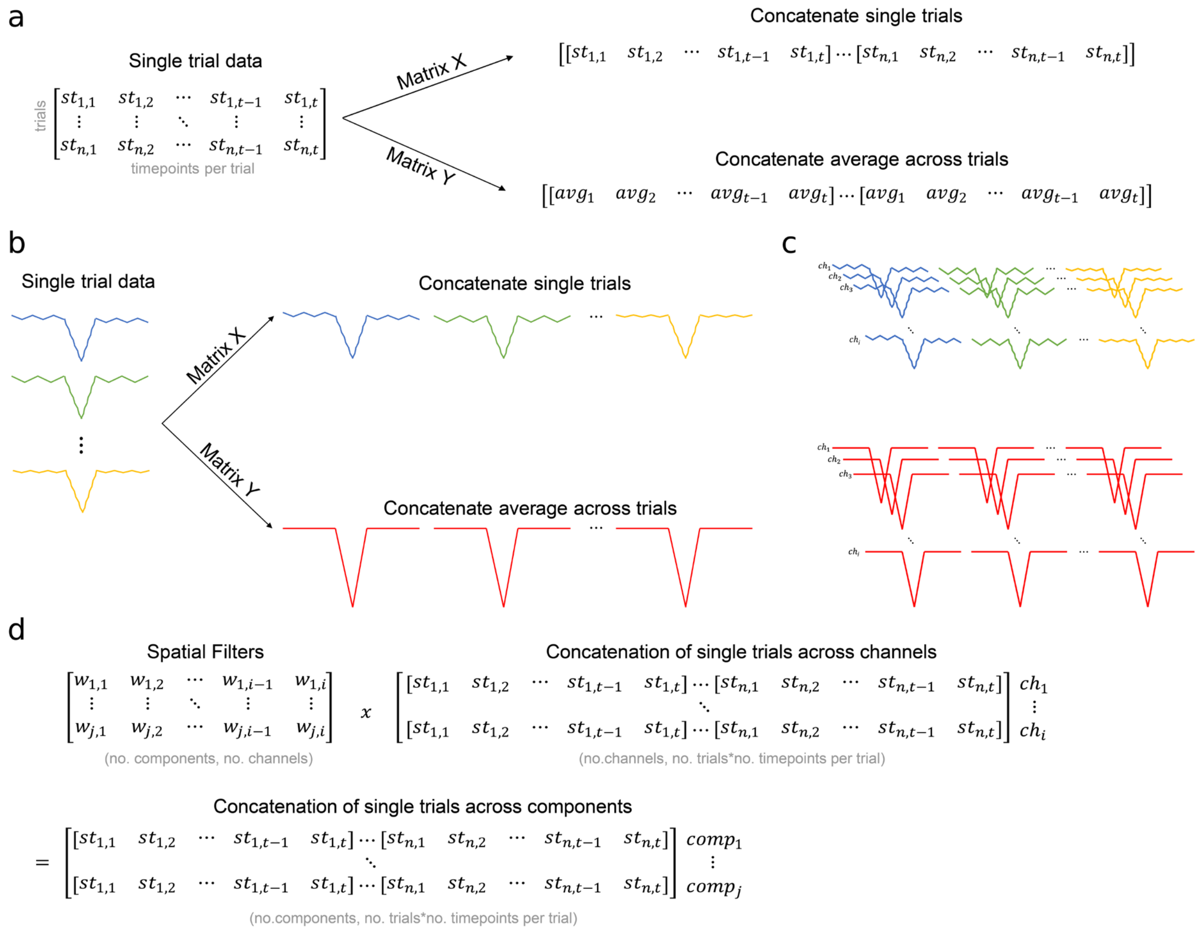

Figure S3: Panel a) shows the way in which single-trial data is used to construct matrices X and Y based upon single channel data. Here, matrix X is constructed by concatenating all single trials in a row, and matrix Y is constructed by averaging across all single trials for this channel, and then concatenating this average signal repeatedly until the number of averages is equal to the total number of single trials. Panel b) represents the same information as a), though in this case the information is shown in a schematic form graphically. Panel c) then shows how the approach shown in a) and b) generalises to multi-channel data (note that CCA only works on multi-channel data). Panel d) demonstrates how the outputs from CCA (spatial filters or weights) are applied to the original data to form components. Note in this case panel d) shows the data structure for CCA-SEP, where the spatial filters are applied to the single trial data. However, for CCA-cardiac the spatial filters are applied to the raw data prior to any epoching, and in this case the data is of shape (no. channels, total no. timepoints in the data) and the components therefore of shape (no. components, total no. timepoints in the data).

5. Single participant time-courses

The single participant plots in this section supplement the group-level results of the main manuscript. Figure S4 depicts the effect of artefact removal methods on the cardiac artefact and Figure S5 shows the SEP time courses with respect to the same artefact removal methods, both for a single participant (sub-010).

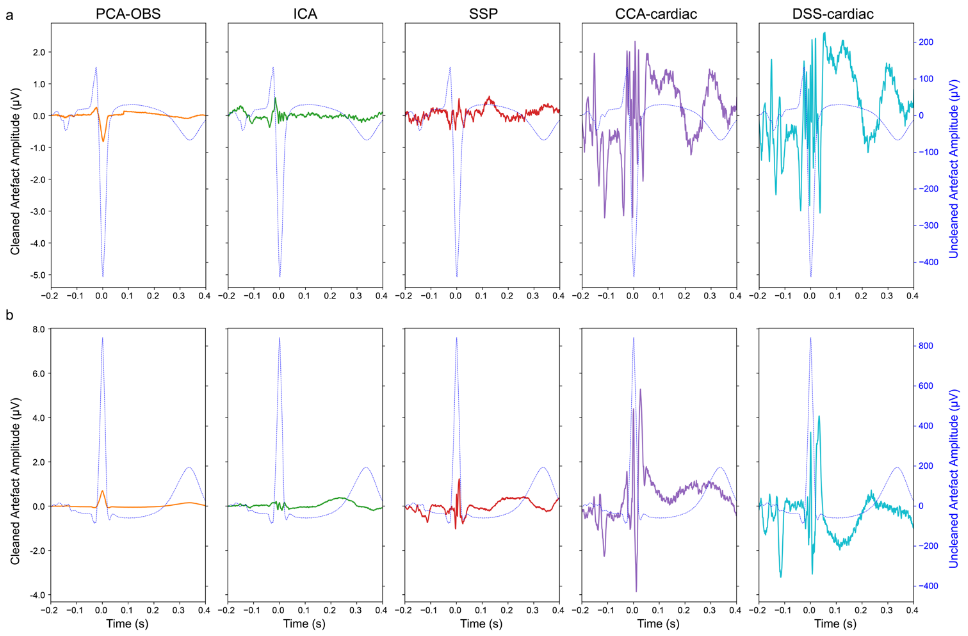

Figure S4: Single-participant (sub-010) cardiac artefact time-courses in the cervical spinal cord (a) and the lumbar spinal cord (b) after application of different cleaning methods, with artefact time-course for Uncleaned data in the background (blue trace).

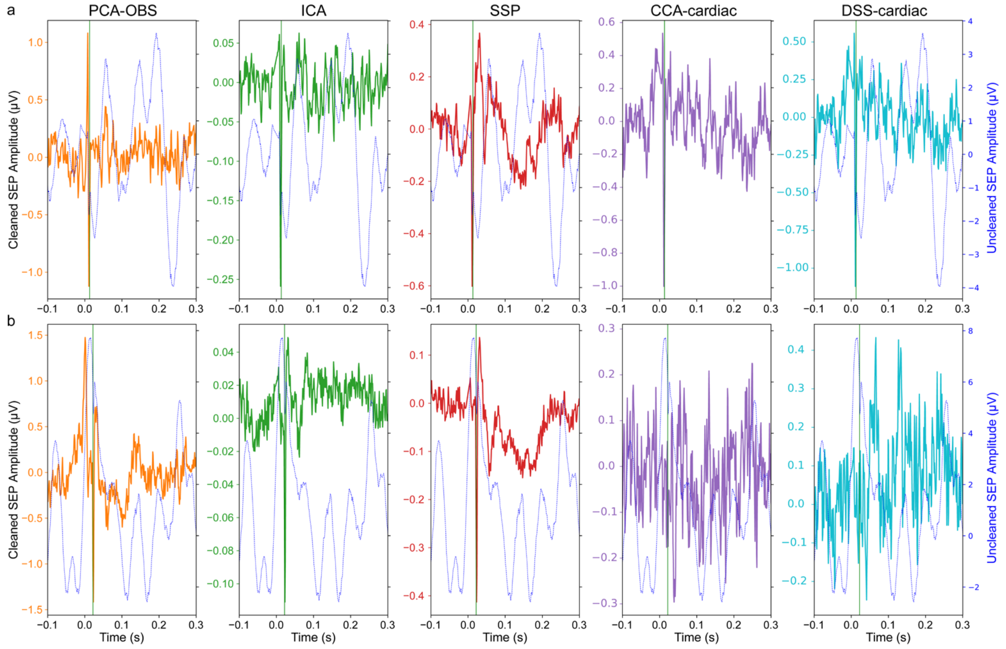

Figure S5: Single-participant (sub-010) SEP time course in the cervical spinal cord (a) and the lumbar spinal cord (b) after application of different cleaning methods, with SEP time-course for Uncleaned data in the background (blue trace). The green line in each plot indicates the expected response latency of the N13 (cervical spinal cord) or the N22 (lumbar spinal cord).

6. Statistics relating to SNR, RI and INPSR for cardiac artefact correction

For the RI, INPSR and SNR results, repeated-measures ANOVAs were performed to determine the effect of each artefact removal method separately for each metric (RI: 1x5 rm-ANOVA, INPSR: 1x5 rm-ANOVA and SNR: 1x6 rm-ANOVA) at each spinal level (cervical and lumbar spinal cord), resulting in a total of six different ANOVAs. This revealed significant differences between the methods ($p<0.05$) in terms of the SNR, RI and INPSR at each spinal level. To directly follow this up with post-hoc tests for head-to-head methods comparisons, after taking the difference between the respective methods, one sample permutation t-tests were performed, with the corrected p-values reported in Table S3.

Table S3: p-values from post-hoc one-sample permutation t-tests testing between which methods there are significant differences at the cervical and lumbar spinal levels with respect to residual intensity (RI), improved normalised power spectrum ratio (INPSR) and signal-to-noise ratio (SNR). Significant p-values (two-tailed after correction for multiple comparisons) are shaded green; please refer to Table 1 in the main manuscript for directionality.

	**Cervical Spinal Cord**			**Lumbar Spinal Cord**		
**Methods Compared**	**RI**	**INPSR**	**SNR**	**RI**	**INPSR**	**SNR**
Uncleaned vs. PCA-OBS	-	-	<0.0001	-	-	0.0002
Uncleaned vs. ICA	-	-	<0.0001	-	-	0.0010
Uncleaned vs. SSP	-	-	<0.0001	-	-	<0.0001
Uncleaned vs. CCA-cardiac	-	-	<0.0001	-	-	0.0045
Uncleaned vs. DSS-cardiac	-	-	<0.0001	-	-	0.0018
PCA-OBS vs. ICA	0.5060	<0.0001	0.0281	0.0421	<0.0001	0.9915
PCA-OBS vs. SSP	<0.0001	<0.0001	0.0002	0.0433	<0.0001	0.0011
PCA-OBS vs. CCA-cardiac	<0.0001	1.0000	0.9628	<0.0001	0.5872	0.9349
PCA-OBS vs. DSS-cardiac	<0.0001	0.5317	0.8865	<0.0001	0.7221	0.3625
ICA vs. SSP	<0.0001	<0.0001	0.0833	<0.0001	<0.0001	0.0126
ICA vs. CCA-cardiac	<0.0001	<0.0001	0.1908	<0.0001	<0.0001	0.9967
ICA vs. DSS-cardiac	<0.0001	<0.0001	0.2348	<0.0001	<0.0001	0.3253
SSP vs. CCA-cardiac	<0.0001	<0.0001	0.0098	<0.0001	<0.0001	0.3830
SSP vs. DSS-cardiac	<0.0001	<0.0001	0.0113	<0.0001	<0.0001	<0.0001
CCA-cardiac vs. DSS-cardiac	0.0263	0.0010	0.8581	0.9993	0.0931	0.2955

7. Ranking of the cardiac artefact removal methods

The ranking of each artefact removal method in terms of the RI, INPSR and SNR can be seen in Table S4, alongside the overall score. Based on this overall score, for both the cervical and lumbar spinal cord, ICA and SSP were the top performing methods.

Table S4: The ranking from first (1) to fifth (5) for each method in a) the cervical spinal cord after median nerve stimulation and b) the lumbar spinal cord after tibial nerve stimulation for the RI, INPSR and SNR. The overall score shown is the weighted average of the rankings across the three evaluated metrics, with the top two performing methods highlighted in green (a lower score indicates better overall performance).

**a)**	***Cervical Spinal Cord***			
	**RI**	**INPSR**	**SNR**	**Overall Score**
PCA-OBS	1	4	5	5.5
ICA	2	1	2	3.5
SSP	3	2	1	4.5
CCA-cardiac	4	4	4	8
DSS-cardiac	5	3	3	8
**b)**	***Lumbar Spinal Cord***			
	**RI**	**INPSR**	**SNR**	**Overall Score**
PCA-OBS	2	4	4	6
ICA	1	1	3	3
SSP	3	2	1	4.5
CCA-cardiac	4	3	2	6.5
DSS-cardiac	5	5	5	10

8. Results and statistics relating to CoV and SNR for signal enhancement

The group-average single trial coefficient of variation and signal-to-noise ratio after applying the signal enhancement methods CCA-SEP and DSS-SEP to Uncleaned, ICA-cleaned and SSP-cleaned data can be seen in Table S5.

Table S5: Single-trial coefficient of variation (CoV; lower values indicate better performance) and signal-to-noise ratio (SNR; higher values indicate better performance) results before enhancement, and after CCA-SEP or DSS-SEP have been applied on either Uncleaned, ICA-cleaned or SSP-cleaned data. Each value represents the group mean ± standard error of the mean.

	Median Nerve Stimulation	Tibial Nerve Stimulation
Single-trial coefficient of variation (CoV)		
Uncleaned	1.45 ± 0.08	2.17 ± 0.08
ICA	0.46 ± 0.02	0.38 ± 0.04
SSP	0.49 ± 0.02	0.64 ± 0.09
Uncleaned + CCA-SEP	0.49 ± 0.02	0.53 ± 0.05
ICA + CCA-SEP	0.45 ± 0.02	0.36 ± 0.02
SSP + CCA-SEP	0.47 ± 0.02	0.49 ± 0.05
Uncleaned + DSS-SEP	0.45 ± 0.02	0.44 ± 0.06
ICA + DSS-SEP	0.43 ± 0.02	0.31 ± 0.02
SSP + DSS-SEP	0.47 ± 0.02	0.37 ± 0.03
Signal-to-noise ratio (SNR)		
Uncleaned	2.45 ± 0.33	1.70 ± 0.23
ICA	11.18 ± 1.14	5.90 ± 0.83
SSP	14.37 ± 1.32	9.92 ± 1.24
Uncleaned + CCA-SEP	19.65 ± 1.69	17.90 ± 1.97
ICA + CCA-SEP	19.49 ± 1.88	9.53 ± 1.32
SSP + CCA-SEP	22.59 ± 2.11	18.59 ± 2.19
Uncleaned + DSS-SEP	11.43 ± 1.47	8.42 ± 1.74
ICA + DSS-SEP	10.42 ± 1.19	4.44 ± 0.76
SSP + DSS-SEP	14.03 ± 1.61	6.66 ± 1.53

As described in the methods, where significant (p<0.05) differences were uncovered by the repeated-measures ANOVAs, post-hoc one-sample permutation t-tests were performed after first subtracting the difference between the relevant conditions - the results can be seen in Table S6

Table S6: p-values from post-hoc one-sample permutation t-tests after median and tibial nerve stimulation with respect to signal-to-noise ratio (SNR) and coefficient of variation (CoV). Significant p-values (two-tailed after correction for multiple comparisons) are shaded green, and '–' indicates post-hoc testing was not performed as the repeated-measures ANOVA did not reveal significant differences between conditions. Refer to Table S5 for directionality.

	Median Nerve Stimulation		Tibial Nerve Stimulation	
**Methods Compared**	**CoV**	**SNR**	**CoV**	**SNR**
Uncleaned vs. Uncleaned + CCA-SEP	<0.0001	<0.0001	<0.0001	<0.0001
Uncleaned vs. Uncleaned + DSS-SEP	<0.0001	0.0003	<0.0001	0.0044
Uncleaned + CCA-SEP vs. Uncleaned + DSS-SEP	0.0176	0.0002	0.0110	<0.0001

ICA vs. ICA + CCA-SEP	-	<0.0001	-	<0.0001
ICA vs. ICA + DSS-SEP	-	0.7473	-	0.0385
ICA + CCA-SEP vs. ICA + DSS-SEP	-	<0.0001	-	<0.0001
SSP vs. SSP + CCA-SEP	-	<0.0001	0.0180	<0.0001
SSP vs. SSP + DSS-SEP	-	0.7219	<0.0001	0.0222
SSP + CCA-SEP vs. SSP + DSS-SEP	-	<0.0001	<0.0001	<0.0001
Uncleaned + CCA-SEP vs. ICA + CCA-SEP	0.0020	0.9611	<0.0001	<0.0001
Uncleaned + CCA-SEP vs. SSP + CCA-SEP	0.0085	0.0025	0.0082	0.3017
ICA + CCA-SEP vs. SSP + CCA-SEP	0.1580	0.0010	<0.0001	<0.0001
Uncleaned + DSS-SEP vs. ICA + DSS-SEP	-	-	0.0111	0.0037
Uncleaned + DSS-SEP vs. SSP + DSS-SEP	-	-	0.0778	0.4243
ICA + DSS-SEP vs. SSP + DSS-SEP	-	-	0.0035	0.0852

9. SEP time courses after the application of CCA-SEP and DSS-SEP

The plots depicted in Figure S6 demonstrate the similarities of the SEPs in both the cervical and lumbar spinal cord after the application of CCA-SEP, while Figure S7 shows the same in relation to DSS-SEP. This means that even in the absence of pre-cleaning of the cardiac artefact, it is possible to obtain high quality SEPs for further analysis.

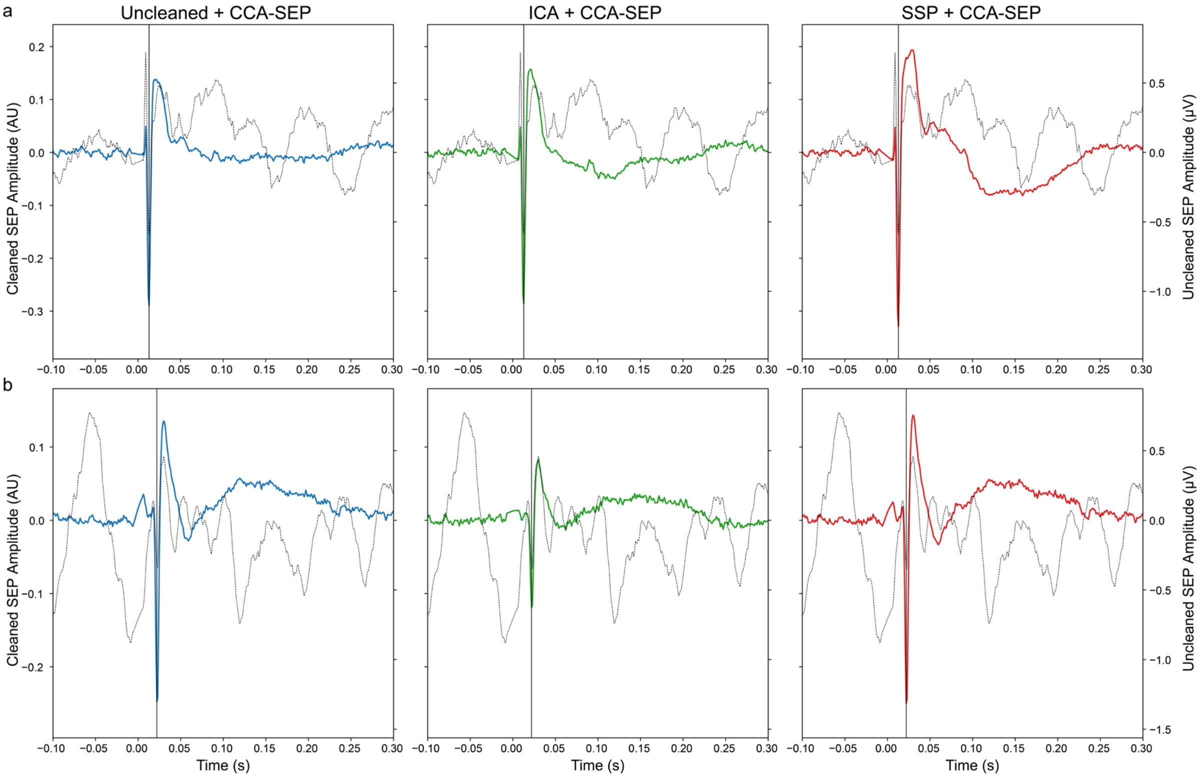

Figure S6: Depiction of the grand average somatosensory evoked potential, for all participants and all trials, after additional processing via the application of CCA-SEP in the cervical (a) and lumbar (b) spinal cord. The left scale bar refers to the coloured traces in each plot after CCA-SEP has been applied. The right scale bar refers to the inset Uncleaned data (grey). The black line indicates the expected latency of the negative potential for median nerve stimulation (13ms) or tibial nerve stimulation (22ms).

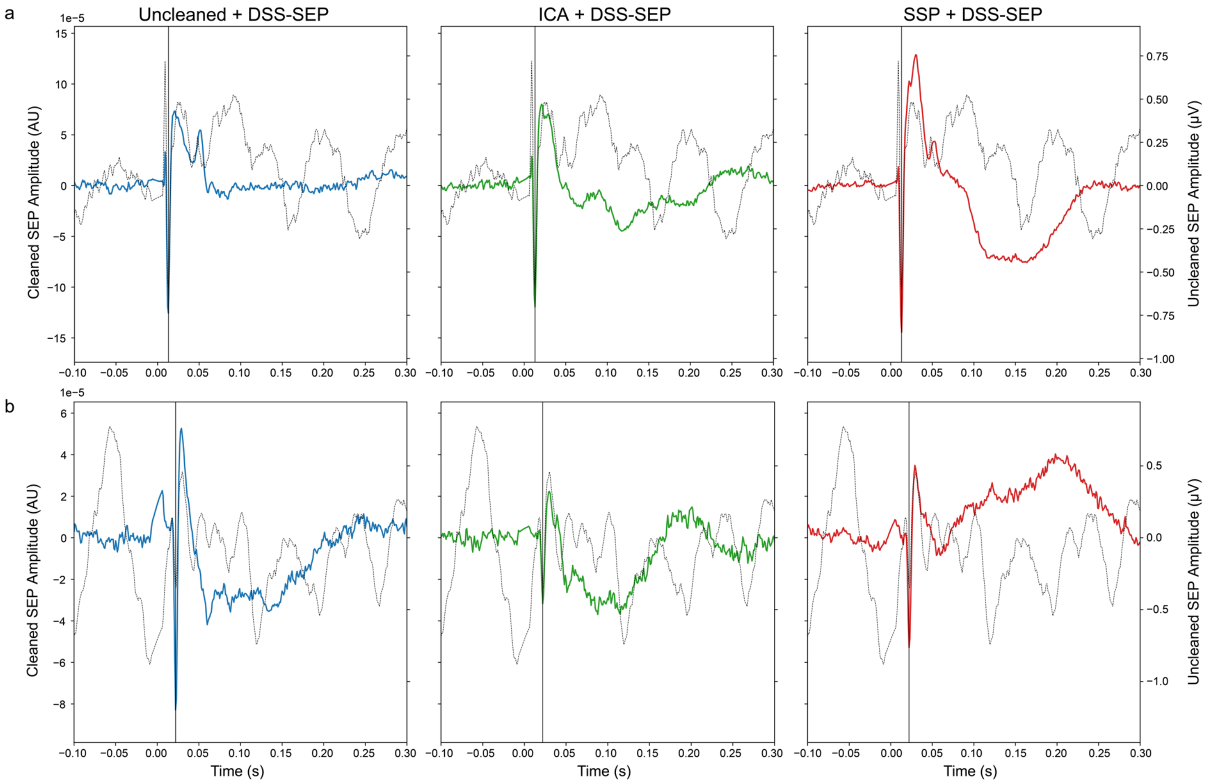

Figure S7: Depiction of the grand average somatosensory evoked potential, for all participants and all trials, after additional processing via the application of DSS-SEP in the cervical (a) and lumbar (b) spinal cord. The left scale bar refers to the coloured traces in each plot after DSS-SEP has been applied. The right scale bar refers to the inset Uncleaned data (grey). The black line indicates the expected latency of the negative potential for median nerve stimulation (13ms) or tibial nerve stimulation (22ms).

10. The ability of CCA-SEP and DSS-SEP to study single trial activity

A key motivation for testing CCA-SEP and DSS-SEP was to determine their ability to enable the study of SEPs at the single trial level. To illustrate this, here we depict cervical spinal cord data from a single participant in response to median nerve stimulation (with the red triangle indicating the expected N13 latency). Figure S8a shows the improvements that can be achieved in robustness of single trial responses after cardiac artefact cleaning alone, with the best visual improvement seen in relation to data cleaned using SSP. Figure S8b demonstrates the additional impact of CCA-SEP on the single-trial responses; here it is clear that robust single trial responses can be obtained using CCA-SEP, even in the absence of dedicated cleaning of the cardiac artefact. Figure S8c demonstrates the impact of DSS; here there is no clear advantage of using DSS-SEP in addition to ICA and SSP cleaning of the cardiac artefact.

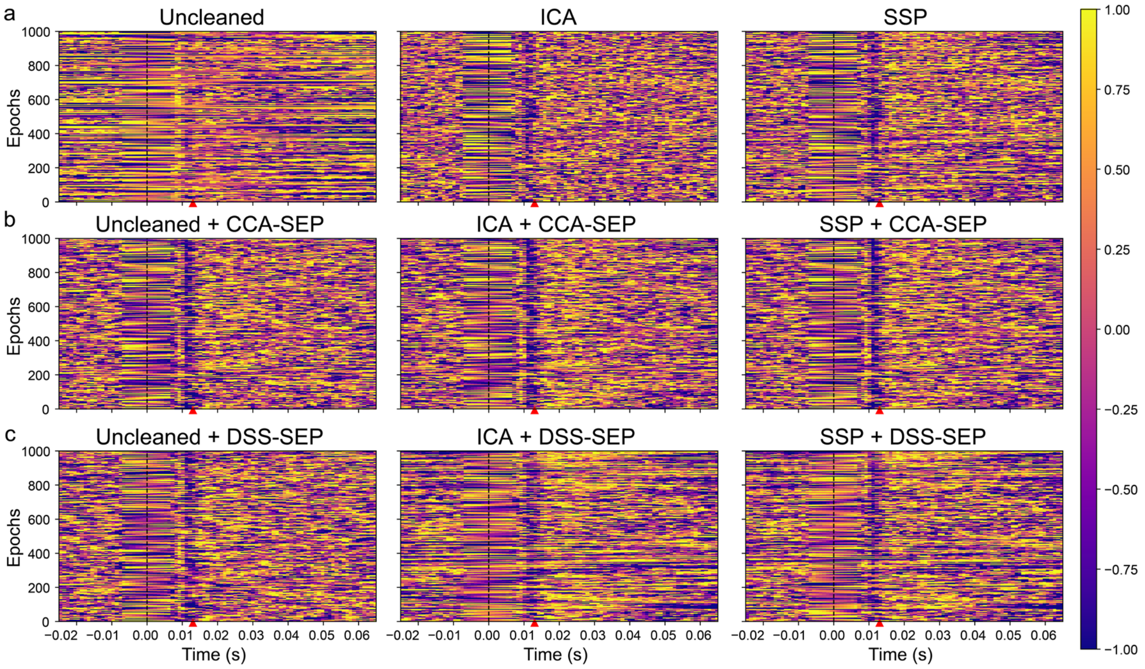

Figure S8: Single trial plots for a single participant (sub-006) whereby each line in a plot is a different trial, created using 1/2 of the available trials in response to median nerve stimulation. Each column shows a different artefact correction method (Uncleaned, ICA, SSP) and each row shows a different signal enhancement method (no enhancement, CCA-SEP enhancement, DSS-SEP enhancement). a) Data prior to the application of CCA-SEP or DSS-SEP in cervical channel SC6. b) Data after CCA-SEP for component 1. c) Data after DSS-SEP for component 1. Each trial has undergone a z-score transformation to allow for comparison. The red triangle marks the canonical expected latency of the N13.
